# Supplementary material for: Programming conformational cooperativity to regulate allosteric protein-oligonucleotide signal transduction
Source: Nat Commun. 2023 Aug 14;14:4898. doi: 10.1038/s41467-023-40589-z (PMC10425332; doi:10.1038/s41467-023-40589-z)
Supplement: Supplementary file 1 — Supplementary Information [file 41467_2023_40589_MOESM1_ESM.pdf]

Programming Conformational Cooperativity to Regulate Allosteric Protein-Oligonucleotide Signal Transduction for Biocomputing

Yuan Liang[1,2†], Yunkai Qie[3,4,5,6†], Jing Yang[2], Ranfeng Wu[1], Shuang Cui[1], Yuliang Zhao[3,4,5], Greg J Anderson[7], Guangjun Nie[3,4,5], Suping Li[3,4,5]*, Cheng Zhang[1]*

[1]School of Computer Science, Key Lab of High Confidence Software Technologies, Peking University, Beijing 100871, China

[2]School of Control and Computer Engineering, North China Electric Power University, Beijing 102206, China

[3]CAS Key Laboratory for Biomedical Effects of Nanomaterials & Nanosafety, CAS Center for Excellence in Nanoscience, National Center for Nanoscience and Technology, Beijing 100190, China

[4]University of Chinese Academy of Sciences, Beijing 100049, China

[5]GBA Research Innovation Institute for Nanotechnology, Guangzhou 510530, China

[6]Department of Urology, Tianjin Institute of Urology, The Second Hospital of Tianjin Medical University, Tianjin 300211, China

[7]QIMR Berghofer Medical Research Institute, Royal Brisbane Hospital, QLD 4029, Australia

†These authors contributed equally to this work.

Table of contents

Supplementary Figures 1-26

S1. Illustrations of the CAST strategy..3

S2. Designs and optimizations of AST operations triggered by thrombin.............4

S2.1 Designs of the AST triggered by thrombin..4

S2.2 Optimizations of the AST operations triggered by thrombin..........................6

S3. Designs and optimizations of AST operations triggered by streptavidin.......10

S3.1 Designs of the AST operations triggered by streptavidin............................10

S3.2 Optimizations of the AST operations triggered by streptavidin..................12

S4. Designs and results of the AST operations triggered by PDGF-BB..............16

S4.1 Designs of the AST operations triggered by PDGF-BB..............................16

S4.2 Results of the AST operations triggered by PDGF-BB...............................18

S5. Triggering AST with saturated protein concentrations..................................19

S6. Designs and results of cooperative regulation of the allosteric signal transducer...20

S7. Designs of the OR logic operations based on the CAST strategy..................23

S8. Designs and optimizations of the AND logic operations based on CAST strategy...24

S8.1 Designs of the AND logic operations...25

S8.2 Optimizations of the AND logic operations..26

S9. The cascading circuit based on the CAST strategy.......................................27

S10. Designs and results of single-trigger-site CAST operations........................31

S10.1 Designs and results of the single-trigger-site CAST operations triggered by thrombin...31

S10.2 Designs and results of the single-trigger-site CAST operations triggered by PDGF-BB...33

S11. Using CAST to regulate GFP gene expression..37

S11.1 Designs of the allosteric transducer to control GFP gene expression.........37

S11.2 Cooperative regulation allosteric transducer to regulate GFP gene expression...41

S12. Using CAST to regulate PLK1 gene expression..43

S12.1 Designs of the AST to regulate PLK1 gene expression........................43

S12.2 Two input logical operations to cooperatively regulates PLK1 gene based on CAST strategy...45

S13. Potential application scenarios of the CAST system....................................47
S14. DNA devices triggered in intracellular and extracellular..............................48
Supplementary Tables 1-11
Table 1. Comparisons of the CAST and other molecule signal transduction methods..49
Table 2. Comparisons of the CAST and the current ASO gene regulation methods..49
Table 3. DNA systems for the extracellular triggering bioengineers....................50
Table 4. DNA based gene regulations with outside cellular triggering................50
Table S1. DNA sequence of the basic allosteric signal transduction system........51
Table S2. DNA sequence of change the length of regulator T2............................51
Table S3. DNA sequence of change the length of hairpin H................................52
Table S4. DNA sequence of the AST triggered by streptavidin...........................52
Table S5. DNA sequence of the AST triggered by PDGF-BB.............................53
Table S6. DNA sequences of CAST using regulators T1 and T2.........................54
Table S7. OR logic operations sequences..55
Table S8. AND logic operations sequences..55
Table S9. Cascade circuit sequences..56
Table S10. DNA sequences of CAST to regulate GFP gene expression..............57
Table S11. DNA sequences of CAST to regulate PLK1 gene expression............58
Table S12. DNA sequences of the single-trigger-site CAST triggered by thrombin..59
Table S13. DNA sequences of the single-trigger-site CAST triggered by PDGF-BB...60
References..61

Supplementary Figures

S1. Illustrations of the CAST strategy.

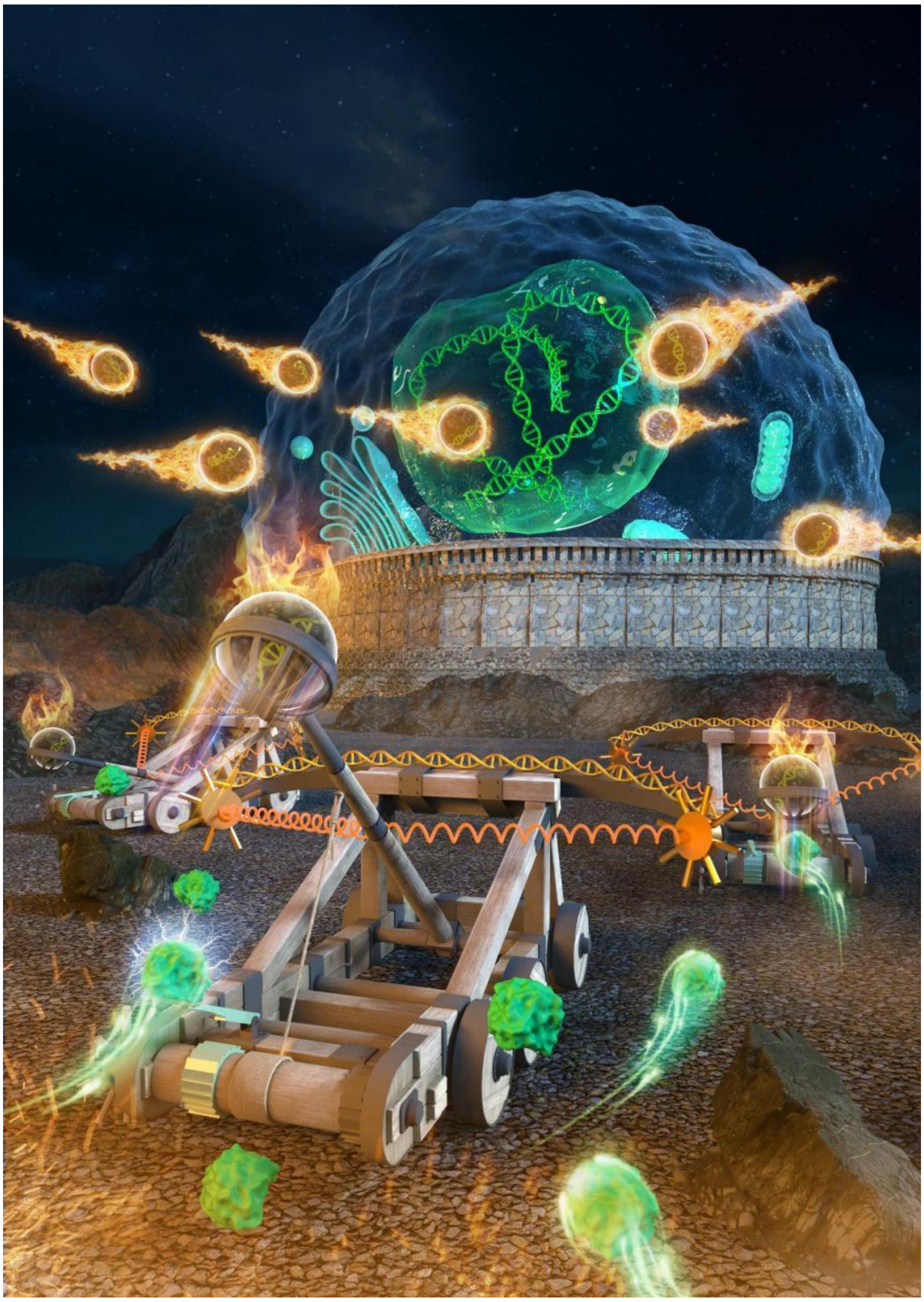

Supplementary Fig. 1 | Illustrations of the CAST strategy.

S2. Designs and optimizations of AST operations triggered by thrombin

S2.1 Designs of the AST operations triggered by thrombin

The signal translator triggered by thrombin is assembled by four DNA strands: A, B ,C and D. The 3' and 5' ends of strand A and strand B are designed with thrombin aptamer sequences with the lengths of 29 nt and 15 nt, respectively, and the complementary domains of DNA C are both 18 bp in length. The length of domain T1 is 3 nt, that of domains T2 is 12 nt, and that of domain H is 12 nt. In particular, the DNA C is a hairpin structure with a 12 nt base complementary hybridization region (domain H). In addition, a 39 nt poly T (domain L) was designed at the 3' end of the D strand to improve the allosteric releasing efficiency. The detailed design is shown in Supplementary Fig. 2 and Table S1-3.

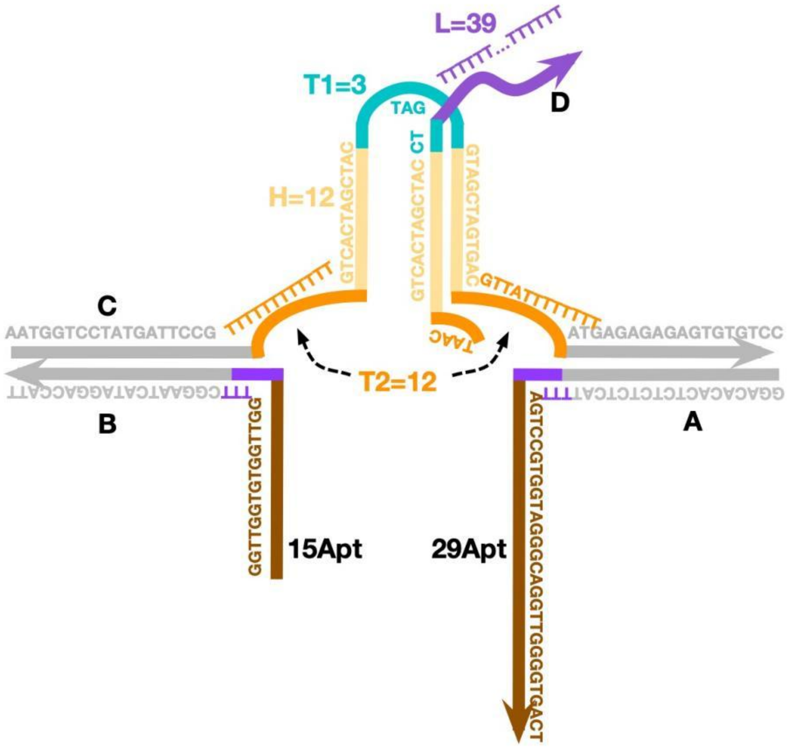

Supplementary Fig. 2 | Schematic diagram of AST using complex CDAB triggered by thrombin.

In the initial state, although the strand C complementary domain H can hybridize to form an intra-hairpin, the thermodynamic state favors hybridization between the strand C and strand D due to the stable state produced by longer hybridization region with 18 nt (Supplementary Fig. 3a). Therefore, strand C preferentially hybridizes with strand D. However, the metastable complex C/D is irritated by conformational changes. To test the binding induced allosteric transduction, we firstly using DNA A5TB to simulate the protein triggering, where two ends of the strand C get close together as shown in Supplementary Fig. 3b. In this state, domains (1) and (2) are adjacent to each other, thus results in a significant increase of the local concentrations of domains (1) and (2), to promote the release of strand D. The NUPACK simulation results verify our designs. In the experimental allosteric regulation, the input proteins are designed to bind to specific allosteric sites on the strands A and B to induce conformational changes and release the corresponding single DNA strand, thus achieving the allosteric signal transduction.

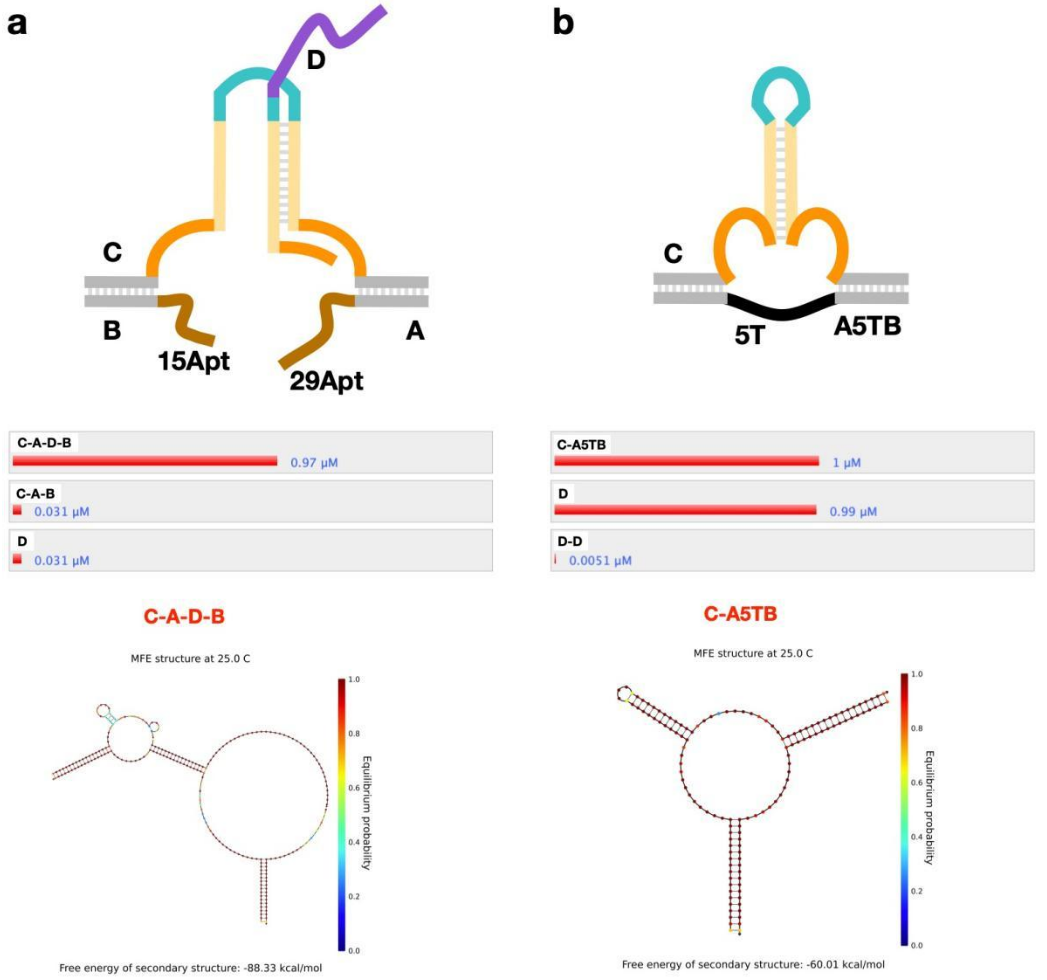

Supplementary Fig. 3 | NUPACK sequence simulation of the AST operations. a,

Schematic diagram and NUPACK simulation results of the assembly of strands C, D, A and B (at 25℃ and 1 μM concentration). **b**, Schematic diagram and NUPACK simulation results of the assembly of strands C, D and A5TB (at 25℃ and 1 μM concentration).

S2.2 Optimizations of the AST operations triggered by thrombin

We explored the optimal structure of the thrombin triggering AST operations by optimizing the lengths of regulator T2 as 4 nt, 8 nt, 12 nt, 16 nt, respectively (Supplementary Fig. 4a and 4b). Upon analysis of fluorescence experiments and PAGE experiments (Supplementary Fig. 4c-4f), better signal noise ratio of the released DNA product was obtained when T2 length was 12 nt. Therefore, we choose 12 nt as the optimal structure for the domain T2 in the following experiments.

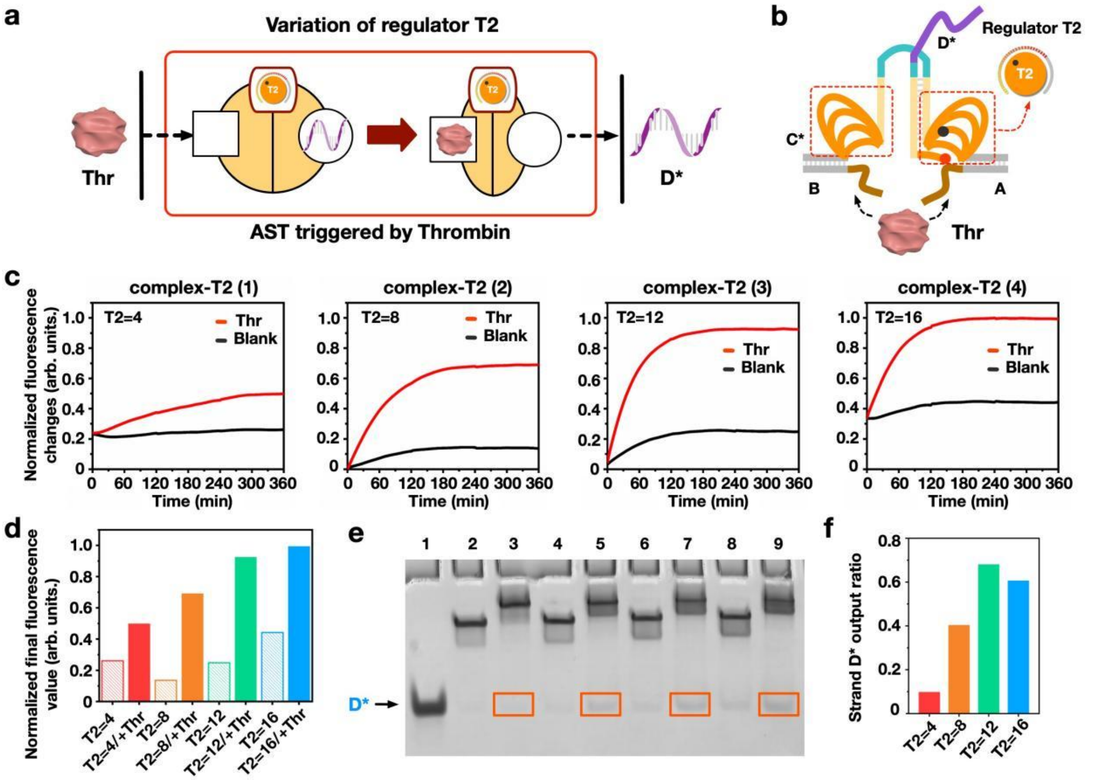

Supplementary Fig. 4 | Regulate the allosteric signal transduction by individually varying the loop lengths of regulator T2. (T1 = 3 nt, H = 11 nt). **a**, Illustrations and **b**, designs of the AST triggered by thrombin with different T2 lengths, respectively. **c**, Fluorescence results of AST triggered by thrombin with different T2 lengths of 4, 8, 12 and 16 nt for complex-T2 (1), (2), (3) and (4). [complex-T2] = 0.5 μM and [Thr] = 1.25 μM. **d**, Quantification of the final fluorescence values of AST triggered by thrombin with different T2 lengths. Native PAGE (12% acrylamide) results **e**, and

analyzed using ImageJ **f**, of AST triggered by thrombin with different T2 lengths. Lane 1: D*, Lane 2: complex-T2 (1), Lane 3: complex-T2 (1) + Thr, Lane 4: complex-T2 (2), Lane 5: complex-T2 (2) + Thr, Lane 6: complex-T2 (3), Lane 7: complex-T2 (3) + Thr, Lane 8: complex-T2 (4), Lane 9: complex-T2 (4) + Thr. [complex-T2] = 0.6 μM and [Thr] = 1.5 μM. Lane 1 was AST output signal controls, and lanes 2, 4, 6, 8 were untriggered controls.

In our design, the length of the complementary region (H domain) between strands C and D is also an important factor that influences AST efficiency. We optimize the domain H of the DNA complex structure of the AST triggered by thrombin (Supplementary Fig. 5). The length of the H domain was used as 10 nt, 11 nt, 12 nt and 13 nt. In the results of the fluorescence experiments and PAGE experiments, better signal noise ratio of the released DNA product was obtained when the length of domain H was 12 nt. Therefore, we choose 12 nt as the optimal structure for the H domain in the following experiments.

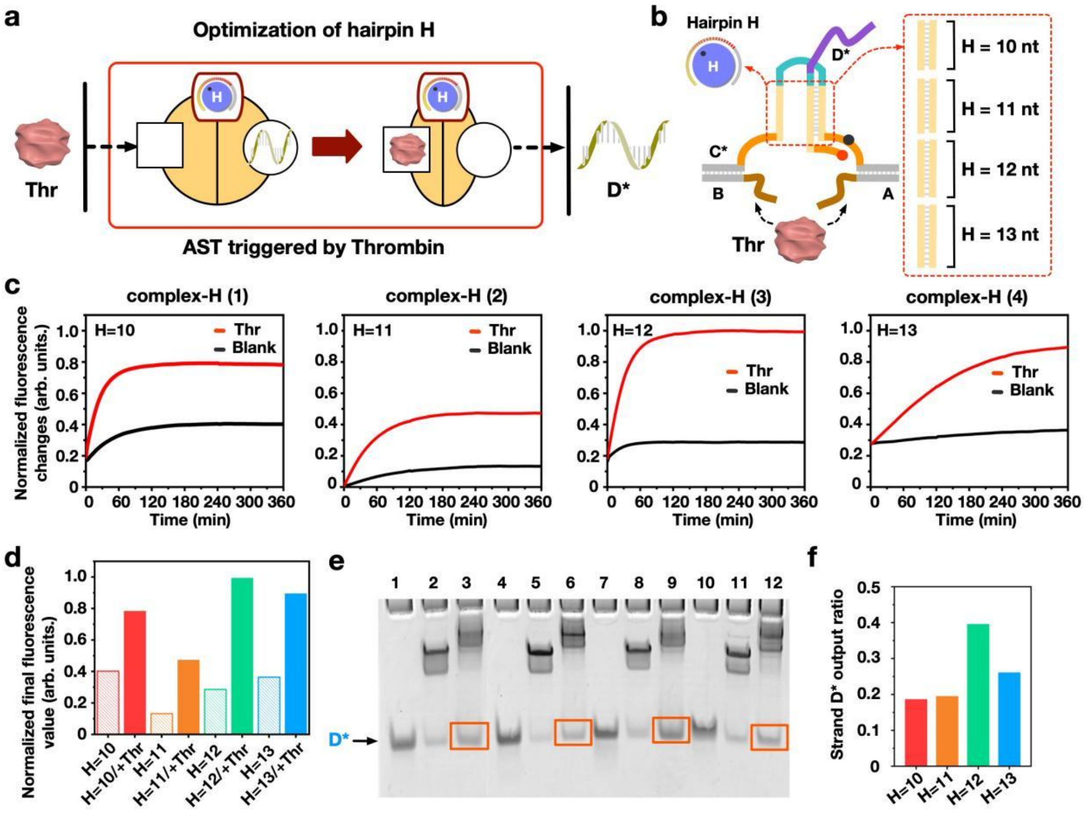

Supplementary Fig. 5 | The length variations of hairpin H to optimize the allosteric signal transduction. (T1 = 3 nt, T2 = 12 nt). **a**, Illustrations and **b**, designs of the AST triggered by thrombin with different H lengths, respectively. **c**,

Fluorescence results of AST triggered by thrombin with different H lengths of 10, 11, 12 and 13 nt for complex-H (1), (2), (3), (4). [complex-H] = 0.5 μM and [Thr] = 1.25 μM. **d**, Quantification of the final fluorescence values of AST triggered by thrombin with different H lengths. Native PAGE (12% acrylamide) results **e**, and analyzed using ImageJ **f**, of AST triggered by thrombin with different H lengths. Lane 1: D2*, Lane 2: complex-H (1), Lane 3: complex-H (1) + Thr, Lane 4: D3*, Lane 5: complex-H (2), Lane 6: complex-H (2) + Thr, Lane 7: D4*, Lane 8: complex-H (3), Lane 9: complex-H (3) + Thr, Lane 10: D5*, Lane 11: complex-H (4), Lane 12: complex-H (4) + Thr. [complex-H] = 0.6 μM and [Thr] = 1.5 μM. Lanes 1-3 and lanes 4-12 are from two different PAGE gels for the same batch of experiments under exactly the same experimental conditions and the gels were processed in parallel. Among them, lanes 1, 4, 7, 10 were AST output signal controls, and lanes 2, 5, 8, 11 were untriggered controls.

On the basis of the optimal structure described above, the optimal concentration of thrombin triggering were determined. As shown in Supplementary Fig. 6a, we explored the optimal thrombin triggering concentration by changing the ratio of thrombin to DNA complex CDAB. Upon increasing thrombin concentration, the complex CDAB combined with more thrombin and formed CAB + Thr complex, and the band moved upward. There was an increasing ejection of strand D, reaching a peak value around 1:2.5. On the other hand, the reaction time of AST operations triggered by thrombin were determined by gel experiments. Supplementary Fig. 6b shows that the release efficiency can exceed 40% even if the reaction time is only five minutes (lane 3), demonstrating the fast efficiency of the allosteric signal transduction. As the time passes, the release of DNA D increases slowly and reaches its peak after about one hour.

On the basis of the optimization described above, we examined the influence of the overall concentration of the reaction system on the model (Supplementary Fig. 6c-6f). There were four reaction systems with concentrations of 0.05 μM, 0.1 μM, 0.2 μM, and 0.5 μM. As a result of the addition of thrombin at the four concentrations, significant changes were observed in fluorescence intensity. Additionally, the higher the reaction system concentration, the greater the fluorescence difference, so in the following fluorescence experiments, we selected a reaction system concentration of 0.5 μM or higher.

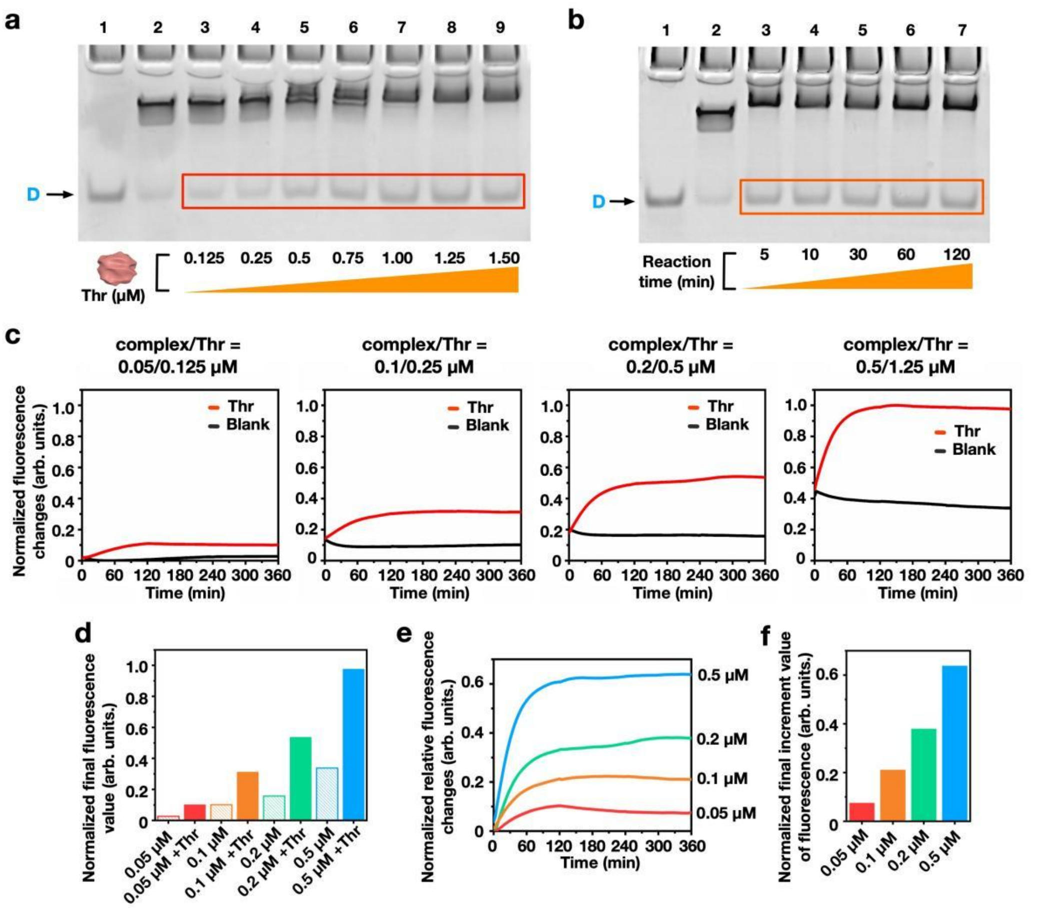

Supplementary Fig. 6 | The optimizations of the experimental conditions of the allosteric signal transduction. **a**, PAGE results of increasing thrombin concentrations to trigger AST. Lane 1: D, Lane 2: complex CDAB, Lanes 3-9: complex CDAB + [Thr] ([complex CDAB] = 0.5 μM, [Thr] as 0.125 μM, 0.25 μM, 0.5 μM, 0.75 μM, 1 μM, 1.25 μM, 1.5 μM, respectively). Lane 1 was AST output signal controls, and lane 2 was untriggered controls. **b**, PAGE results of the reaction time of the AST operations triggered by thrombin. Lane 1: D, Lanes 2-7: CDAB + Thr (Reaction time = 0, 5 min, 10 min, 30 min, 60 min, 120 min, respectively). [complex CDAB] = 0.5 μM. [Thr] = 1.25 μM. Lane 1 was AST output signal controls, and lane 2 was untriggered controls. **c**, Fluorescence results of the AST reaction systems with different concentrations of reactants. (1): [complex CDAB] = 0.05 μM, [Thr] = 0.125 μM; (2): [complex CDAB] = 0.1 μM, [Thr] = 0.25 μM; (3): [complex CDAB] = 0.2 μM, [Thr] = 0.5 μM; (4): [complex CDAB] = 0.5 μM, [Thr] = 1.25 μM. **d**, Histogram comparison of final fluorescence values of AST with different reactant concentrations. **e**, The relative fluorescence variations with different reactant concentrations at each time point for AST. **f**, Histogram of the final increment value of fluorescence results in (**e**).

S3. Designs and optimizations of the AST operations triggered by streptavidin

S3.1 Designs of the AST operations triggered by streptavidin

Based on the AST model mentioned above, the AST operations triggered by streptavidin was also designed. In the AST operations using streptavidin, the DNA complex was designed as: the domain H 11 nt, the domain T 13 nt, and the domain T2 12 nt. In addition, the binding sites that recognizes streptavidin on the DNA A and B strands was changed to biotin. The detailed design is shown in Supplementary Fig. 7 and Table S4.

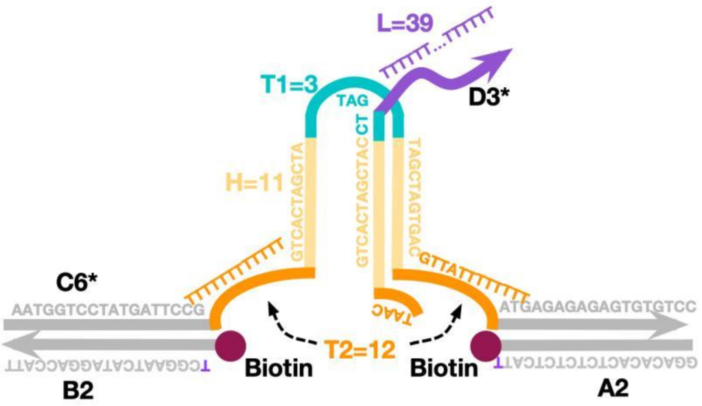

Supplementary Fig. 7 | Schematic diagram of AST operations triggered by streptavidin.

We performed NUPACK simulation for the AST operations triggered by streptavidin. In the initial state, although the strand C6* complementary domain H can hybridize to form an intra-hairpin, the thermodynamic state favors hybridization between the strand C6* and strand D3* due to the stable state produced by longer hybridization region with 18 nt (Supplementary Fig. 8a). Therefore, strand C6* preferentially hybridizes with strand D3*. To test the binding induced allosteric transduction, we firstly using DNA A5TB to simulate the protein triggering, where two ends of the strand C6* get close together as shown in Supplementary Fig. 8b. In this state, domains (1) and (2) are adjacent to each other, thus results in a significant increase of the local concentrations of domains (1) and (2), to promote the release of strand D3*. The NUPACK simulation results verify our designs.

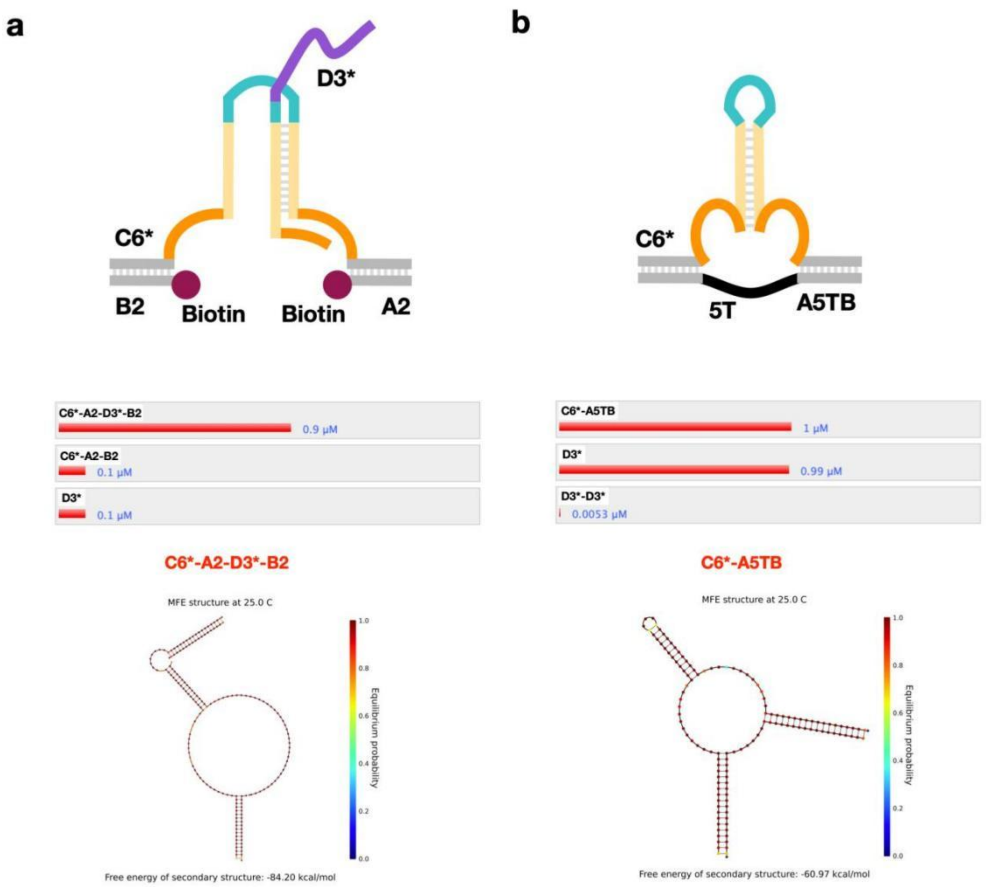

Supplementary Fig. 8 | NUPACK sequence simulation of the AST operations triggered by streptavidin. a, Schematic diagram and NUPACK simulation results of the assembly of strands C6*, D3*, A2 and B2 (at 25℃ and 1 μM concentration). **b**, Schematic diagram and NUPACK simulation results of the assembly of strands C6*, D3* and A5TB (at 25℃ and 1 μM concentration).

S3.2 Optimizations of the AST operations triggered by streptavidin

We explored the optimal structure of the streptavidin triggering AST operations by optimizing the lengths of regulator T2 as 4 nt, 8 nt, 12 nt, 16 nt, respectively (Supplementary Fig. 9a, 9b and Table S2). Upon analysis of fluorescence experiments and PAGE experiments (Supplementary Fig. 9c-9f), better signal noise ratio of the released DNA product was obtained when T2 length was 12 nt. Therefore, we choose 12 nt as the optimal structure for the domain T2 in the following experiments.

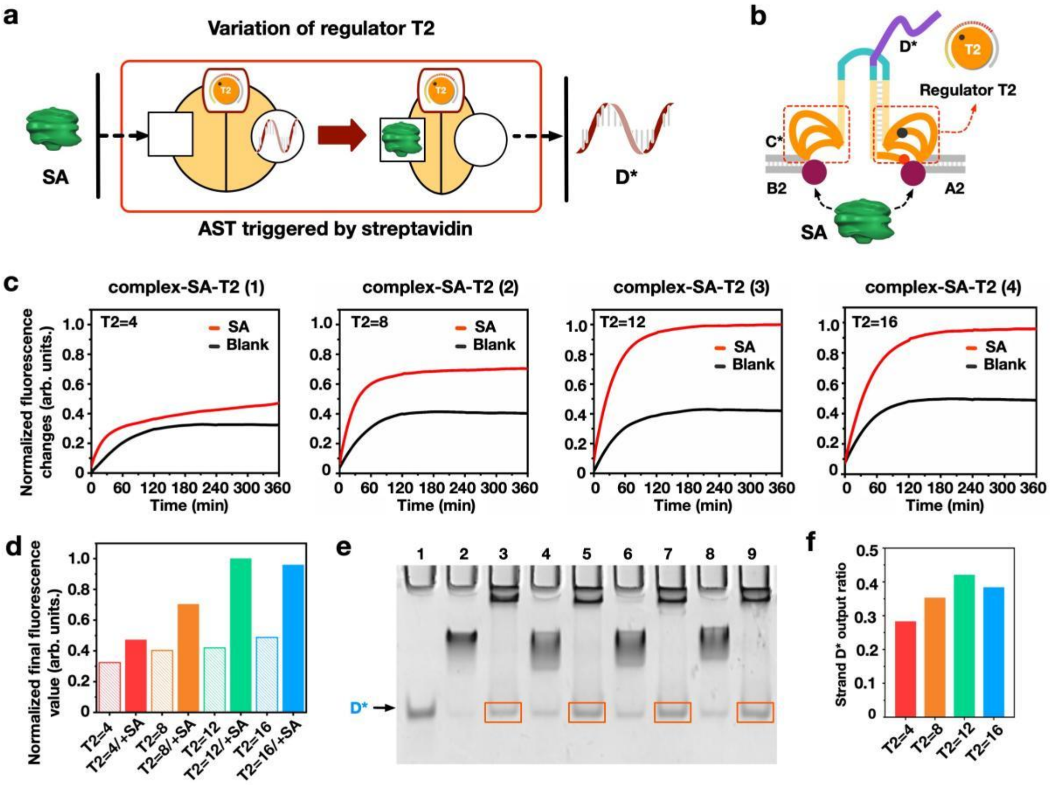

Supplementary Fig. 9 | Regulate the allosteric signal transduction triggered by streptavidin by individually varying the loop lengths of regulator T2. (T1 = 3 nt, H = 11 nt). a, Illustrations and **b**, designs of the AST operations with different T2 lengths, respectively. **c**, Fluorescence results of AST triggered by streptavidin with different T2 lengths of 4, 8, 12 and 16 nt for complex-SA-T2 (1), (2), (3) and (4). [complex-SA-T2] = 0.5 μM and [SA] = 1.25 μM. **d**, Quantification of the final fluorescence values of AST operations. **e**, Native PAGE results and **f**. analyzed using ImageJ. Lane 1: D1*, Lane 1: complex-SA-T2 (1), Lane 2: complex-SA-T2 (1) + SA, Lane 3: complex-SA-T2 (2), Lane 4: complex-SA-T2 (2) + SA, Lane 5: complex-SA-T2 (3), Lane 6: complex-SA-T2 (3) + SA, Lane 7: complex-SA-T2 (4), Lane 8: complex-SA-T2 (4) + SA. [complex-SA-T2] = 0.6 μM and [SA] = 1.5 μM. Lane 1 was AST output signal controls, and lanes 2, 4, 6, 8 were untriggered controls.

In our design, the length of the complementary region (H domain) between strands C and D is also an important factor that influences AST efficiency. We optimize the domain H of the DNA complex structure of the AST triggered by streptavidin (Supplementary Fig. 10 and Table S3). The length of the H domain was used as 10 nt, 11 nt, 12 nt and 13 nt. In the results of the fluorescence experiments and PAGE experiments, better signal noise ratio of the released DNA product was obtained when the length of domain H was 11 nt. Therefore, we choose 11 nt as the optimal structure for the H domain in the following experiments.

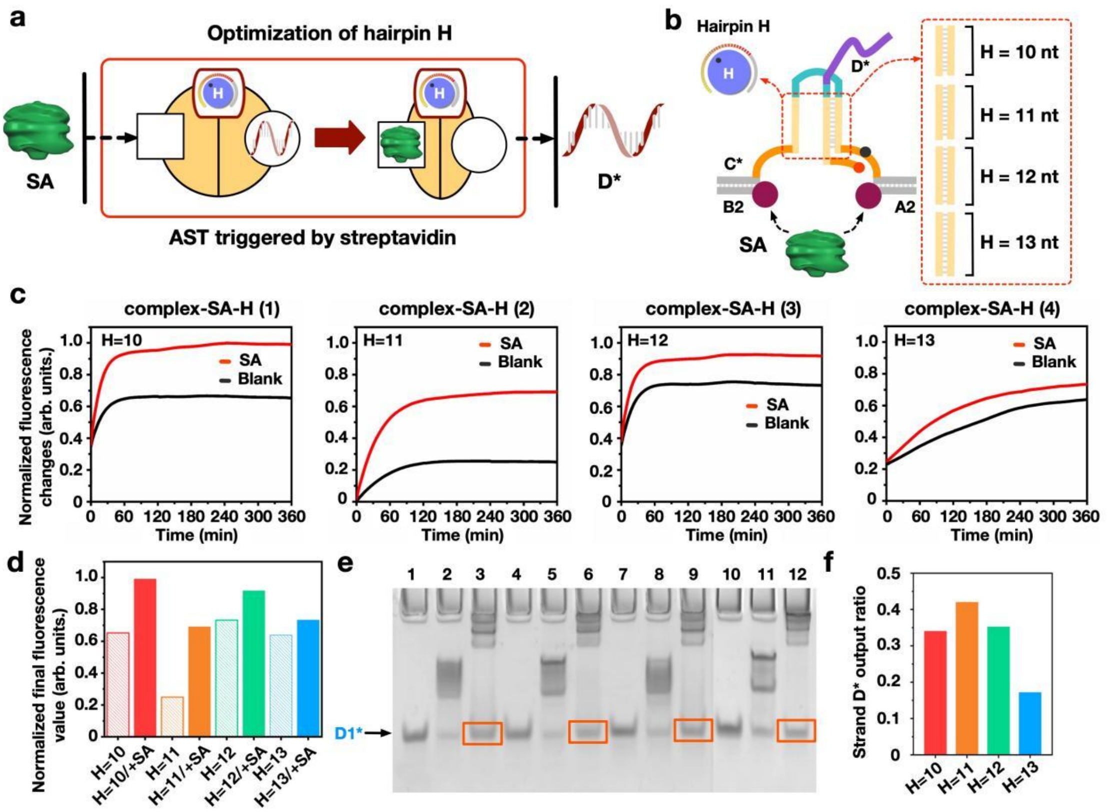

Supplementary Fig. 10 | The length variations of domain H to optimize the AST operations triggered by streptavidin. (T1=3 nt, T2=12 nt). **a**, Illustrations and **b**, designs of the AST triggered by streptavidin with different H lengths, respectively. **c**, Fluorescence results of AST triggered by streptavidin with different H lengths of 10, 11, 12 and 13 nt for complex-SA-H (1), (2), (3), (4). [complex-SA-H] = 0.5 μM and [SA] = 1.5 μM. **d**, Quantification of the final fluorescence values of AST operations. **e**, Native PAGE results and **f**, analyzed using ImageJ Lane 1: D2*, Lane 2: complex-SA-H (1), Lane 3: complex-SA-H (1) + SA, Lane 4: D3*, Lane 5: complex-SA-H (2), Lane 6: complex-SA-H (2) + SA, Lane 7: D4*, Lane 8: complex-SA-H (3), Lane 9: complex-SA-H (3) + SA, Lane 10: D5*, Lane 11:

complex-SA-H (4), Lane 12: complex-SA-H (4) + SA. [complex-SA-H] = 0.6 μM and [SA] = 1.8 μM. Lanes 1-9 and lanes 10-12 are from two different PAGE gels for the same batch of experiments under exactly the same experimental conditions and the gels were processed in parallel. Lanes 1, 4, 7, 10 were AST output signal controls, and lanes 2, 5, 8, 11 were untriggered controls.

On the basis of the optimal structure described above, the optimal concentration of streptavidin triggering were determined. As shown in Supplementary Fig. 11a, we explored the optimal streptavidin triggering concentration by changing the ratio of streptavidin to DNA complex C6*D3*A2B2. Upon increasing thrombin concentration, the complex C6*D3*A2B2 combined with more streptavidin and formed C6*A2B2 + SA, and the band moved upward. There was an increasing ejection of strand D, reaching a peak value around 1:3. On the other hand, the reaction time of AST operations triggered by streptavidin were determined by gel experiments. Supplementary Figure 11b shows the change of release efficiency over time, which proves the efficient and fast release of allosteric signal transduction.

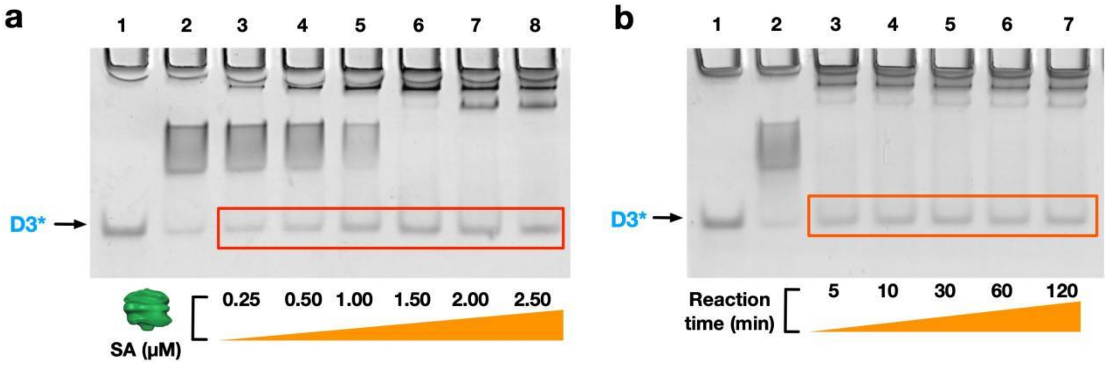

Supplementary Fig. 11 | The optimizations of the experimental conditions of the allosteric signal transduction triggered by streptavidin. a, PAGE results of increasing streptavidin concentrations to trigger AST. Lane 1: D3*, Lane 2: complex C6*D3*A2B2, Lanes 3-9: complex C6*D3*A2B2 + [SA] ([complex C6*D3*A2B2] = 0.5 μM, [SA] as 0.25 μM, 0.5 μM, 1 μM, 1.5 μM, 2 μM and 2.5 μM, respectively). Lane 1 was AST output signal controls, and lane 2 was untriggered controls. **b**, PAGE results of the reaction time of the AST operations triggered by streptavidin. Lane 1: D3*, Lanes 2-7: C6*D3*A2B2 + SA (Reaction time = 0, 5 min, 10 min, 30 min, 60 min, 120 min, respectively). [complex C6*D3*A2B2] = 0.5 μM. [SA] = 1.5 μM. Lane 1 was AST output signal controls, and lane 2 was untriggered controls.

After the completion of all optimization experiments, PAGE and fluorescence experiments were used to verify the optimal AST triggered by streptavidin (Supplementary Fig. 12). In PAGE analysis (Supplementary Fig. 12c), after 2 hour of reaction, it can be seen in lane 5 that translator binds to streptavidin and releases D3* strand. In the fluorescence experiment, the fluorescence value of the translator was compared with that after adding 3 times streptavidin, and it could be seen that the fluorescence value was significantly increased within 2 hours after adding streptavidin to the translator (Supplementary Fig. 12d).

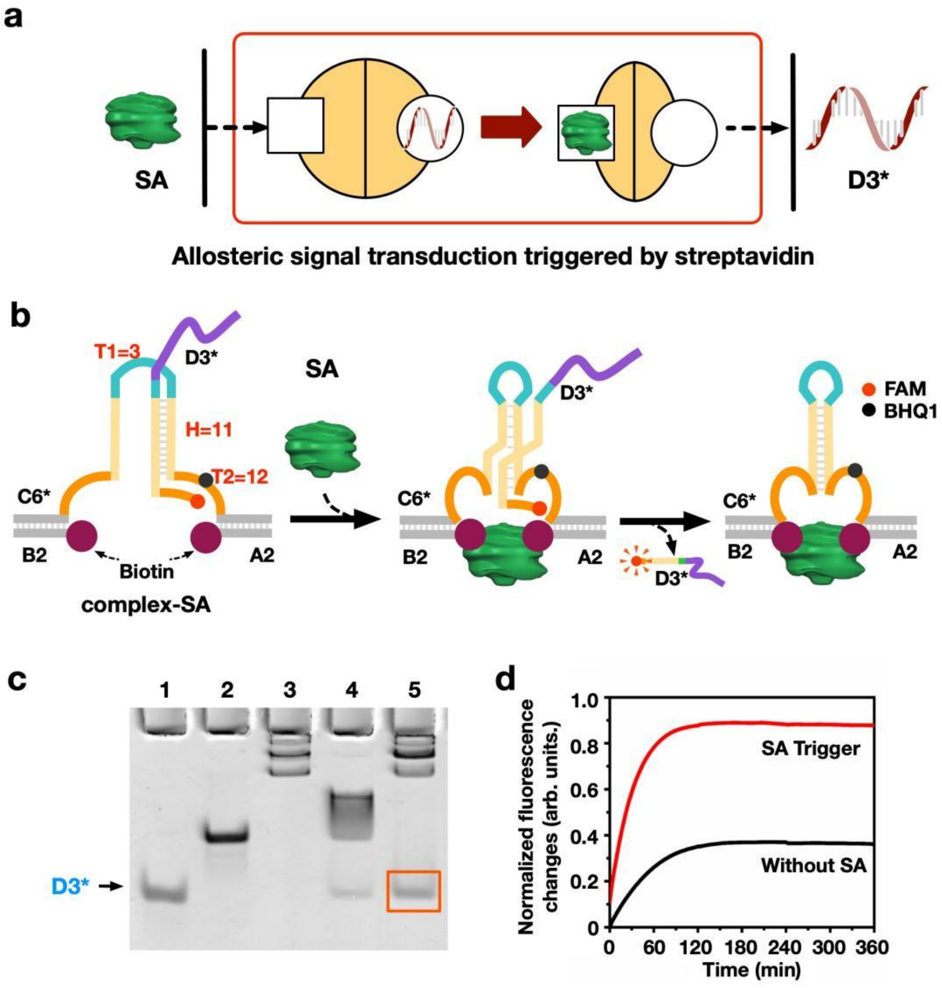

Supplementary Fig. 12 | Allosteric signal transduction triggered by streptavidin. **a**,**b**, Schematic illustration (**a**) and design (**b**) of a basic allosteric signal transduction triggered by streptavidin, respectively. **c**, PAGE gel analysis of the basic allosteric transduction system. Lane 1: D3*; Lane 2: C6*A2B2; Lane 3: C6*A2B2 + SA; Lane 4: C6*D3*A2B2; Lane 5: C6*D3*A2B2 + SA. **d**, Fluorescence output of the basic allosteric transduction system triggered by streptavidin. [DNA complex] = 0.5 μM, [SA] = 1.5 μM.

S4. Designs and results of the AST operations triggered by PDGF-BB

S4.1 Designs of the AST operations triggered by PDGF-BB

The AST operations triggered by PDGF-BB was designed using DNA complex-PDGF, CDA3B3. The length of domain H was 12 nt, that of domain T1 was 3 nt, and that of domain T2 was 12 nt. In addition, aptamers sequences 35Apt that binds PDGF-BB were designed at the ends of DNA A3 and B3. The detailed design is shown in Supplementary Fig. 13 and Table S5.

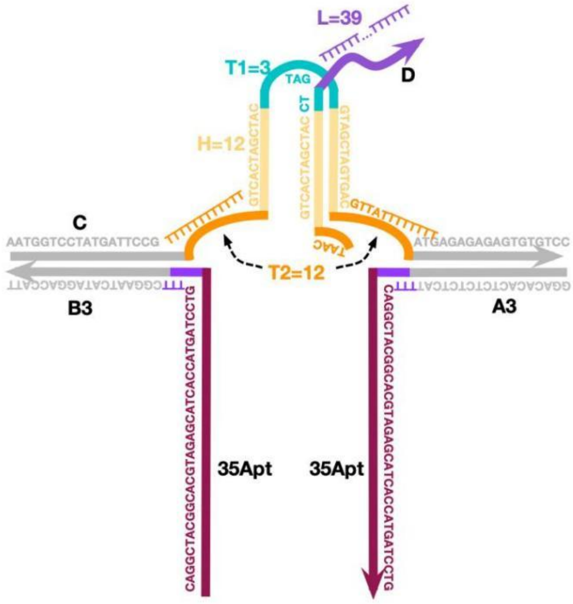

Supplementary Fig. 13 | Schematic diagram of AST operations triggered by PDGF-BB structure sequence.

We performed NUPACK simulation for the AST operations triggered by PDGF-BB. In the initial state, although the strand C complementary domain H can hybridize to form an intra-hairpin, the thermodynamic state favors hybridization between the strand C and strand D due to the stable state produced by longer hybridization region with 18 nt (Supplementary Fig. 14a). Therefore, strand C preferentially hybridizes with strand D. To test the binding induced allosteric transduction, we firstly using DNA A5TB to simulate the protein triggering, where two ends of the strand C get close together as shown in supplementary Fig. 14b. In this state, domains (1) and (2) are adjacent to each other, thus results in a significant increase of the local concentrations of domains (1) and (2), to promote the release of strand D. The NUPACK simulation results verify our designs.

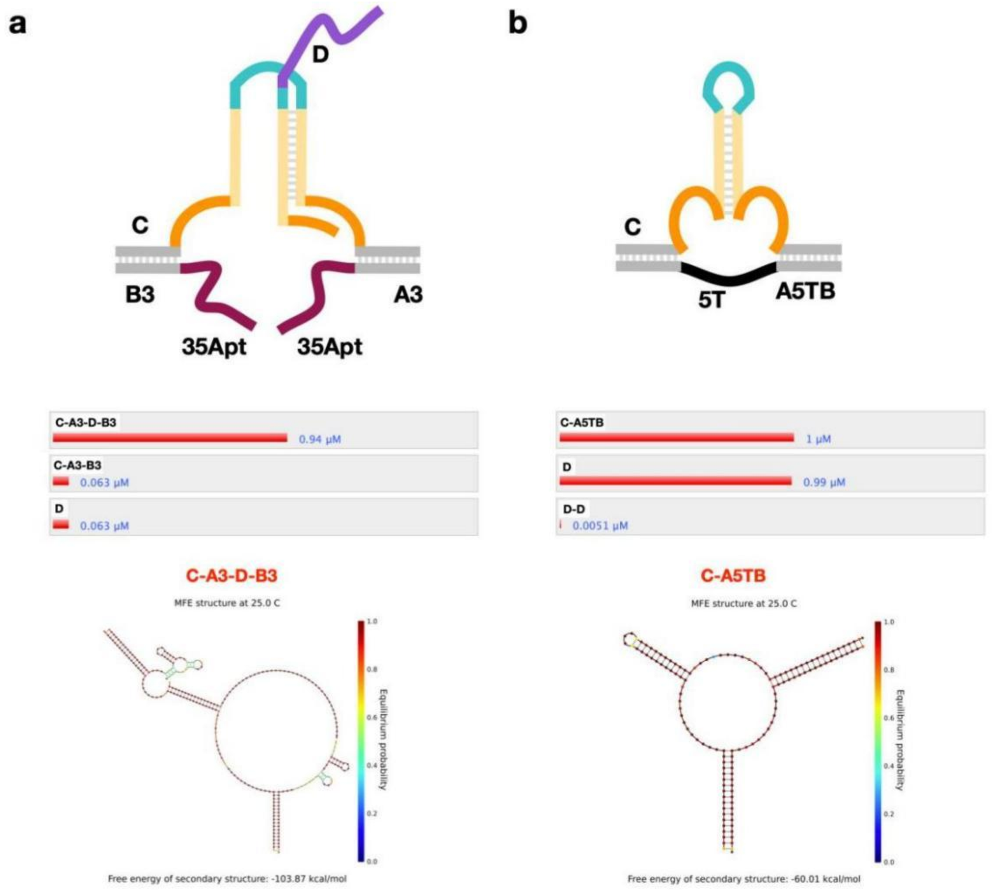

Supplementary Fig. 14 | NUPACK sequence simulation of the AST operations triggered by PDGF-BB. a, Schematic diagram and NUPACK simulation results of the assembly of strands C, D, A3 and B3 (at 25℃ and 1 μM concentration). **b,** Schematic diagram and NUPACK simulation results of the assembly of strands C, D and A5TB (at 25℃ and 1 μM concentration).

S4.2 Results of the AST operations triggered by PDGF-BB

The detailed mechanism of the AST triggered by PDGF-BB model is shown in supplementary Fig. 15a and 15b. With the increasing PDGF-BB, the released D strand gradually increased to the maximum peak (from lanes 2 to 5), and then the release decreased at the highest concentration of PDGF-BB as 1.8 μM (lane 6) (Supplementary Fig. 15c). This may be due to competition induced re-conjugation between PDGF-BB and the DNA aptamer, causing an oversaturation that interferes with the AST effects.

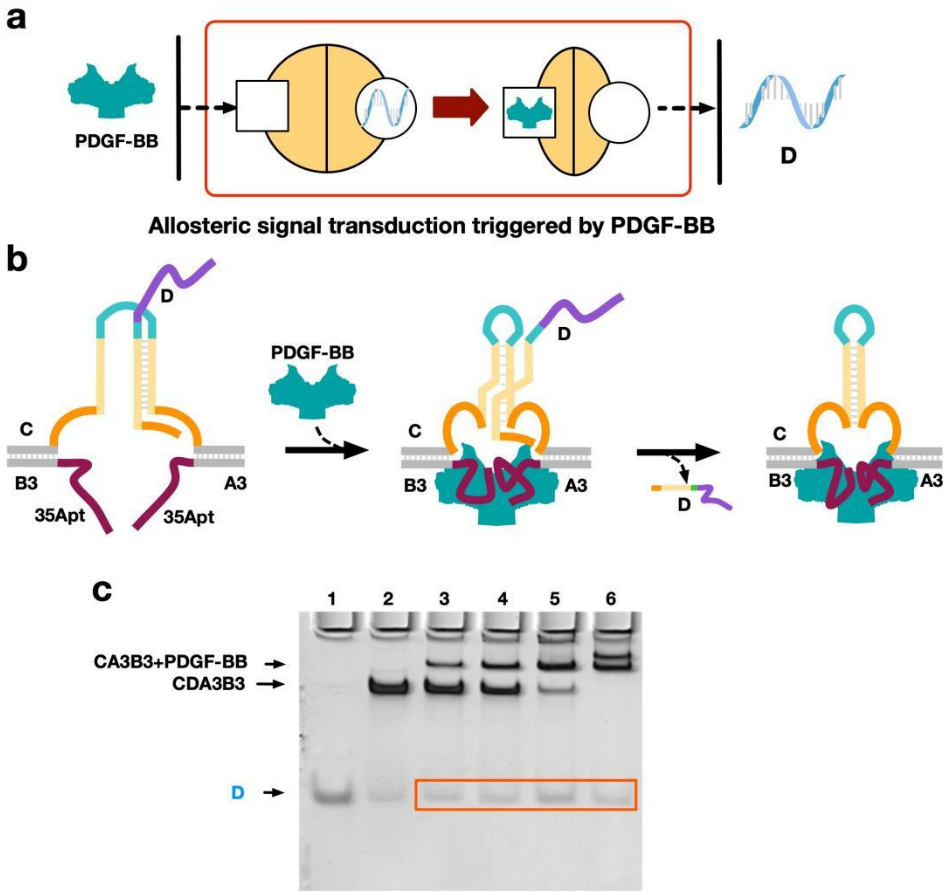

Supplementary Fig. 15 | The results of AST operations triggered by PDGF-BB. a, Schematic diagram of the AST operations triggered by PDGF-BB. **b**, Native PAGE analysis of the AST with different PDGF-BB concentrations. [complex-PDGF]=0.6 μM. Lanes 2-6 were added with PDGF-BB of 0, 0.3, 0.6, 1.2, and 1.8 μM, respectively. Lane 1 was AST output signal controls, and lane 2 was untriggered controls.

S5. Triggering AST with saturated protein concentrations

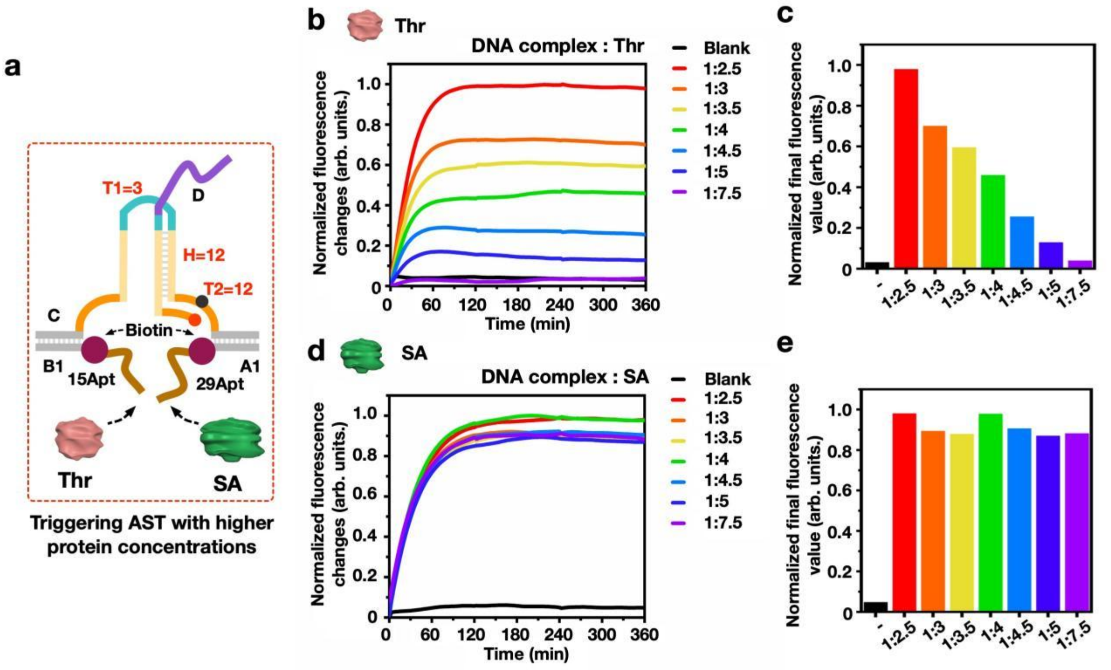

Supplementary Fig. 16 | Triggering AST with saturated protein concentrations of thrombin and streptavidin. a, Designs of trigger AST with saturated protein concentrations of thrombin and streptavidin. **b**, Fluorescence results of the AST triggered by saturated concentrations of thrombin. [complex-or] = 0.5 µM. [Thr] = 0, 1.25 µM, 1.5 µM, 1.75 µM, 2.0 µM, 2.25 µM, 2.5 µM, 3.75 µM. **c**, Histogram of the final increment value of fluorescence results of high concentrations of thrombin. **d**, Fluorescence results of the AST triggered by high concentrations streptavidin. [complex-or] = 0.5 µM. [SA] = 0, 1.25 µM, 1.5 µM, 1.75 µM, 2.0 µM, 2.25 µM, 2.5 µM, 3.75 µM. **e**, Histogram of the final increment value of fluorescence results using saturated concentrations of streptavidin.

S6. Designs and results of cooperative regulation of the allosteric signal transducer

According to the previous experimental results of the AST triggered by thrombin model structure optimization, we kept the domain H constant to 12 nt, and varied the length of regulator T1 and T2 to implement the cooperative regulation of allosteric signal transducers. The detailed design is shown in Supplementary Fig. 17, 18 and Table S6. As shown in Supplementary Fig. 19, fluorescence data results of all 9 kinds of CAST regulations.

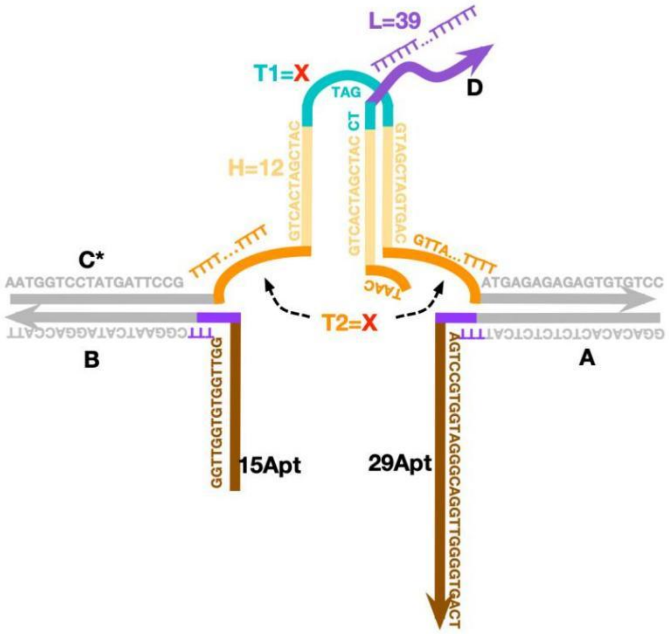

Supplementary Fig. 17 | Schematic diagram of the DNA complex used in CAST operations.

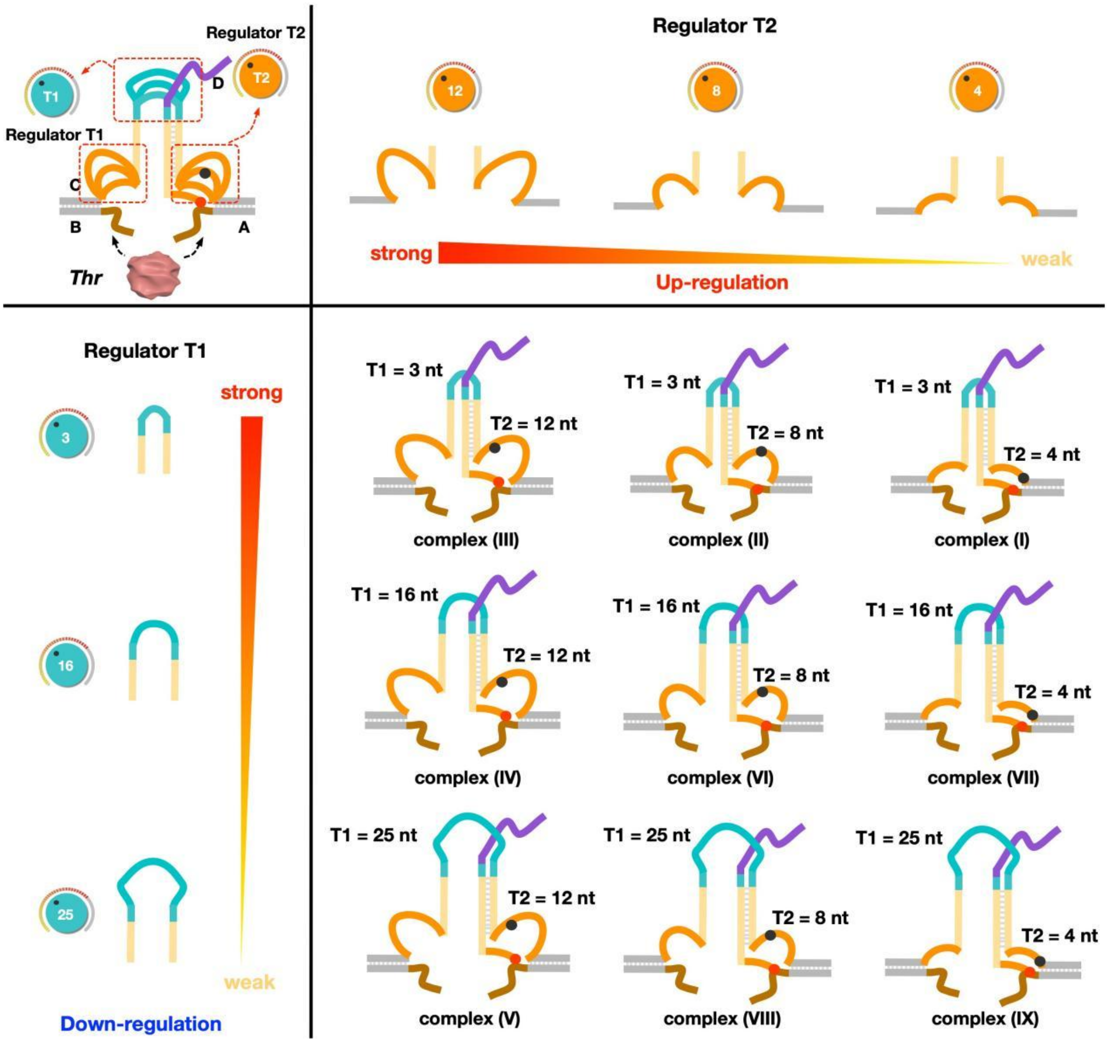

Supplementary Fig. 18 | Structural design of all 9 DNA complexes used in the cooperative regulations of the signal transduction.

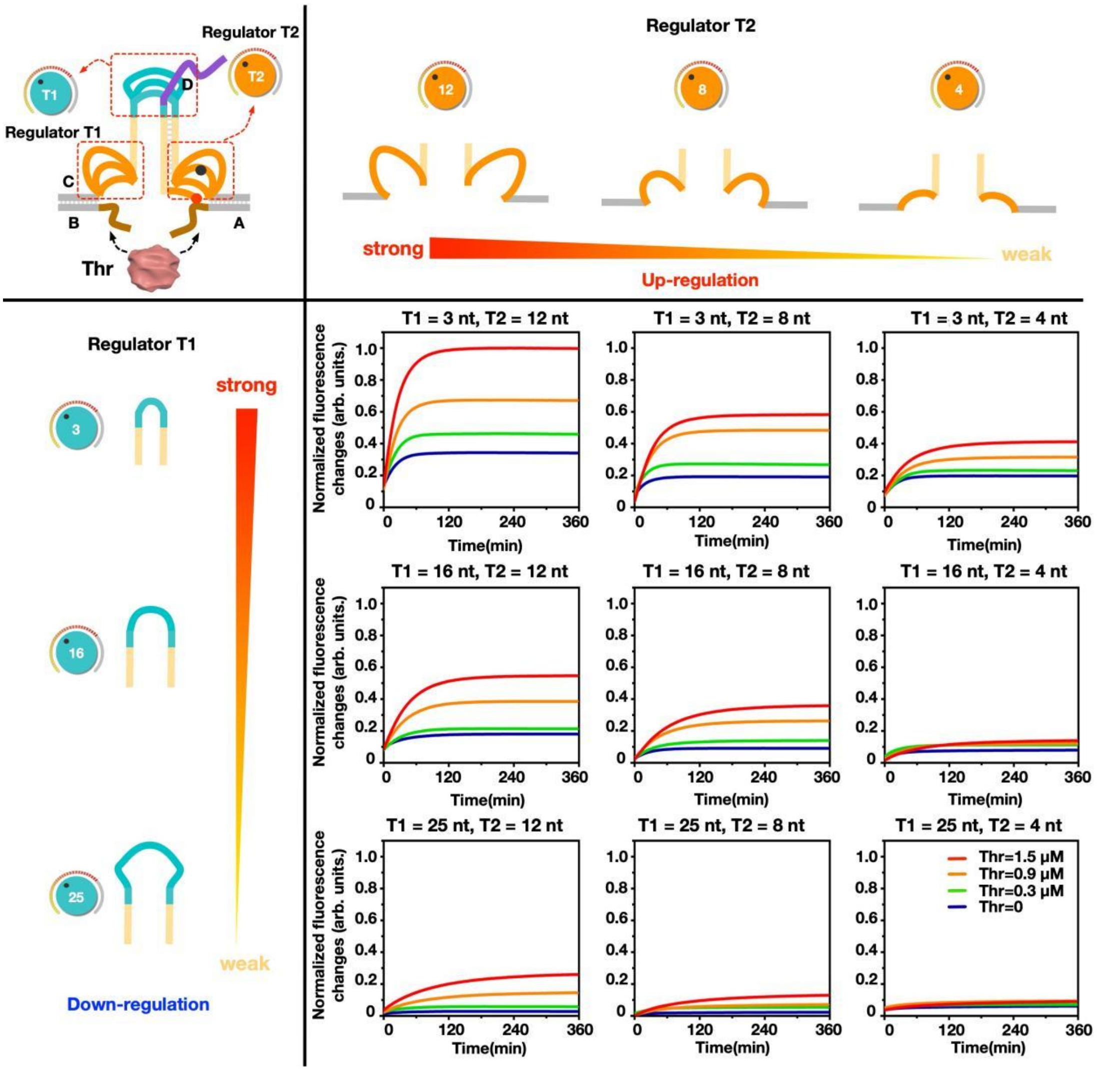

Supplementary Fig. 19 | Fluorescence data results of all 9 kinds of CAST regulations using regulators T1 and T2. [complex] = 0.6 μM. [Thr] = 0, 0.3 μM, 0.9 μM, 1.5 μM.

S7. Designs of the OR logic operations based on the CAST strategy

Based on the CAST model, we use two proteins of thrombin and streptavidin to implement OR gate operation. In the OR gate, the length of domain H was 12 nt, that of domain T1 was 3 nt, and that of domain T2 was 12 nt. In addition, two biotin modifications were designed at the ends of aptamer sequence and the detailed design is shown in Supplementary Fig. 20 and Table S7.

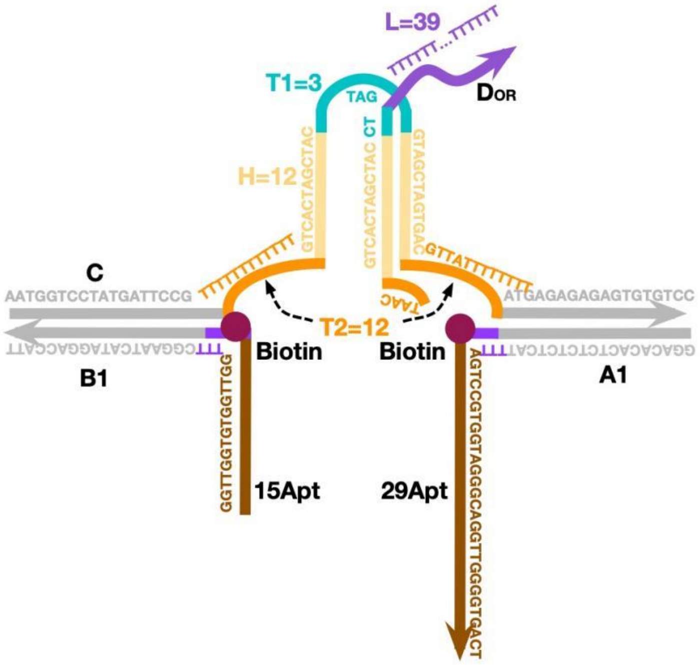

Supplementary Fig. 20 | Schematic diagram of OR gate DNA complex.

S8. Designs and optimizations of the AND logic operations based on CAST strategy

S8.1 Designs of the AND logic operations

In order to realize the design of AND logic gate, we developed a CAST DNA complex with two separated structures: B2C1 and A2C2D$_{AND}$ (Supplementary Fig. 21 and Table S8). DNA A2 and strand B2 respectively modified biotin for streptavidin recognition. Therefore, in the absence of protein input, the DNA complex-and stayed at the state as two separated structures (C1-B2 and C2-A2-D$_{AND}$), without releasing DNA D$_{AND}$. When any one of proteins: thrombin and streptavidin is introduced, two separated structures were still kept for not enough protein binding connection, and no allosteric transduction was performed to release DNA D$_{AND}$. Only in the presence of both proteins of thrombin and streptavidin, both ends of C1-B2 and C2-A2-D$_{AND}$ approach close to each other, thus prompting the release of DNA D$_{AND}$. NUPACK sequence simulation of AND logic gate complex is shown in Supplementary Fig. 22.

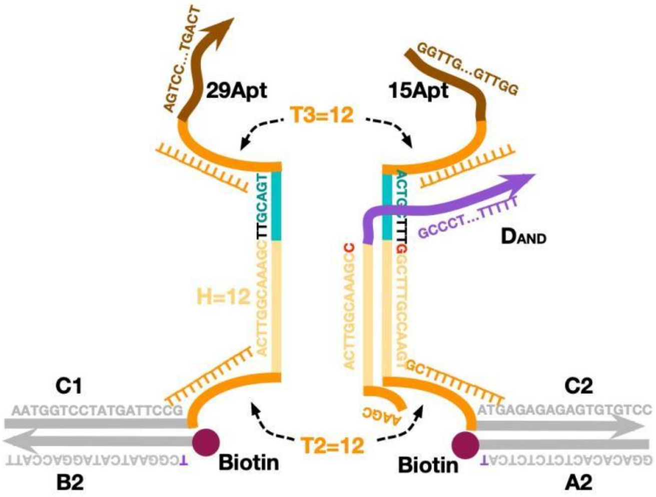

Supplementary Fig. 21 | Schematic diagram of AND gate DNA complex.

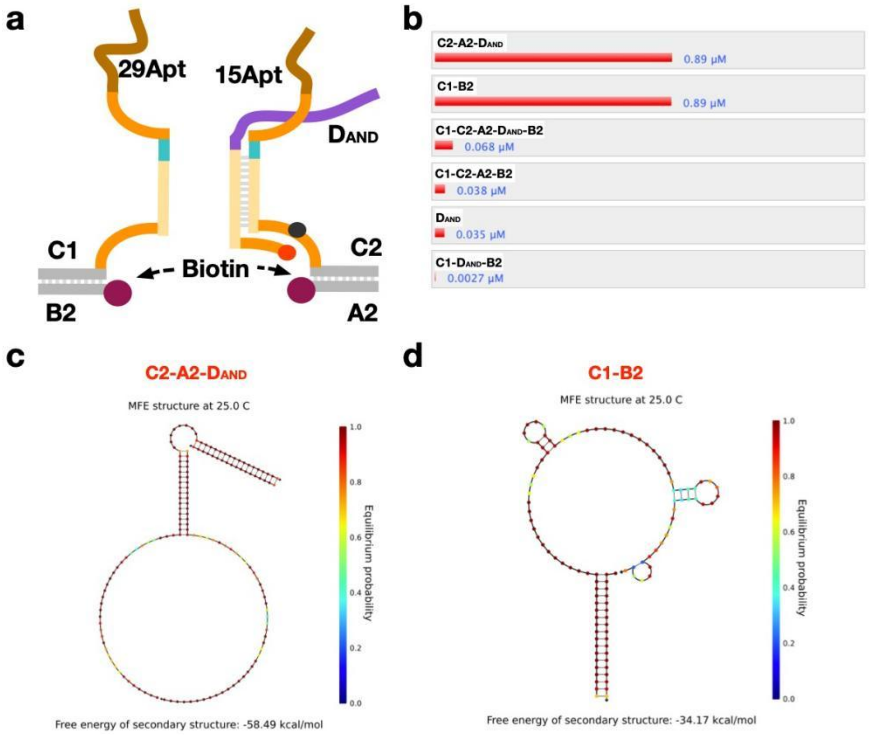

Supplementary Fig. 22 | NUPACK sequence simulation of AND logic gate complex. a, Schematic diagram of NUPACK simulation results of AND logic gate complex. **b**, NUPACK simulation results of four single strands of AND gate complex at 25 ℃ and 1 μM concentration. The secondary structure of **c**, C2-A2-D_{AND} and **d**, C1-B2.

S8.2 Optimizations of the AND logic operations

To optimize the AND logic operation, we designed four kinds of DNA complex structures complex-and (1), (2), (3) and (4), and verified the operations by PAGE experiments (Supplementary Fig. 23 and see Table S8 for sequence design). Specifically, in complex-and (1), the length of domain H was 12 nt, that of domain T2 was 12 nt, and that of domain T3 was 12 nt; in complex-and (2), the length of domain H was 13 nt, that of domain T2 was 12 nt, and that of domain T3 was 12 nt; in complex-and (3), the length of domain H was 18 nt, that of domain T2 was 12 nt, and that of domain T3 was 12 nt, with DNA D_{AND} hybridization length as 13 nt; in complex-and (4), the length of domain H was 17 nt, that of domain T2 was 12 nt, and that of domain T3 was 12 nt, with DNA D_{AND} hybridization length as 12 nt. In the PAGE results, a significant release of DNA D_{AND} can be found only in complex-and (4) as shown in Supplementary Fig. 23. Therefore, we choose the structure of complex-and (4) in the AND logic operations.

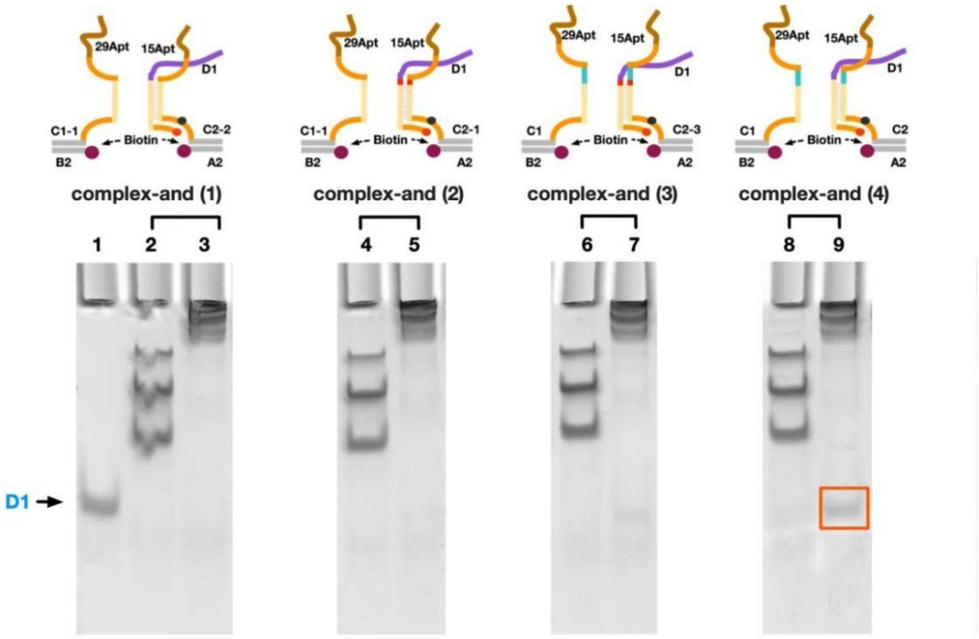

Supplementary Fig. 23 | Regulation of molecular translators by AND logic gate. Lane 1: D_{AND}, Lane 2: complex-and (1), Lane 3: complex-and (1) + Thr + SA, Lane 4: complex-and (2), Lane 5: complex-and (2) + Thr + SA, Lane 6: complex-and (3), Lane 7: complex-and (3) + Thr + SA, Lane 8: complex-and (4), Lane 9: complex-and (4) + Thr + SA. Lanes 1-5 and lanes 6-9 are from two different PAGE gels for the same batch of experiments under exactly the same experimental conditions and the gels were processed in parallel. Lane 1 was AST output signal controls, and lanes 2, 4, 6, 8 was untriggered controls.

S9. The cascading circuit based on the CAST strategy

To further explore the CAST strategy, switchable and cascading allosteric signal transductions has been constructed(Supplementary Fig. 24). In the switchable system, DNA S* and R* binding with the site of toehold 1 and 2 in complex (X) to displace DNA S and R, respectively, thus making complex (X) at "ON" state with dual biotin modifications. In this condition, the "ON" complex (X) can be triggered by streptavidin to release DNA D_{Cd}. In the cascading system, the thrombin is designed to triggered the upstream complex (XI) through the allosteric signal transduction to release of DNA S* (modified with biotin). Then, DNA S* binding with the site of toehold 1 in downstream complex (XII) to displace DNA S, thus making complex (XII) at "ON" state with dual biotin modifications. Only in this condition, the "ON" complex (XII) can be triggered by streptavidin to release DNA D_{Cd}. The sequence design and simulation are shown in Supplementary Fig. 25-27 and Table S9.

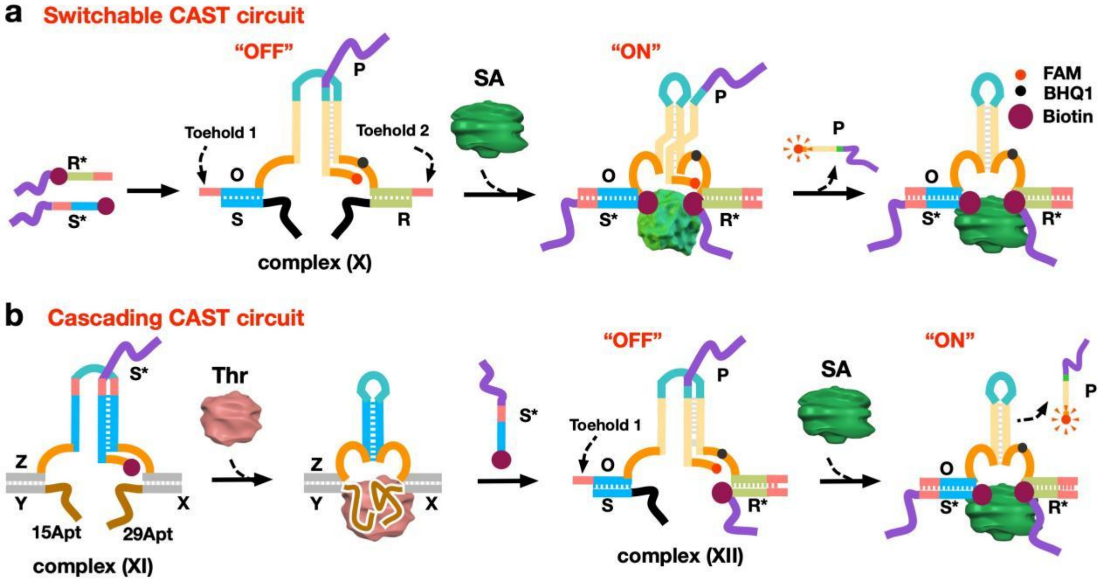

Supplementary Fig. 24 | Schematic diagram of switchable and cascade circuit. Schematic diagram of **a**, switchable AST circuit and **b**, cascading CAST circuit.

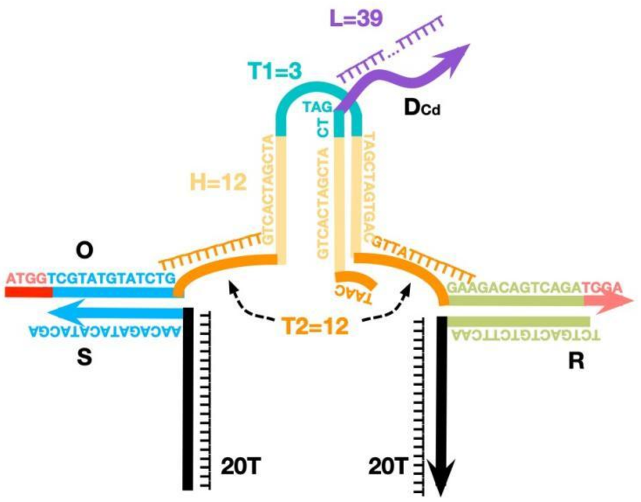

Supplementary Fig. 25 | Schematic diagram of DNA complex (X).

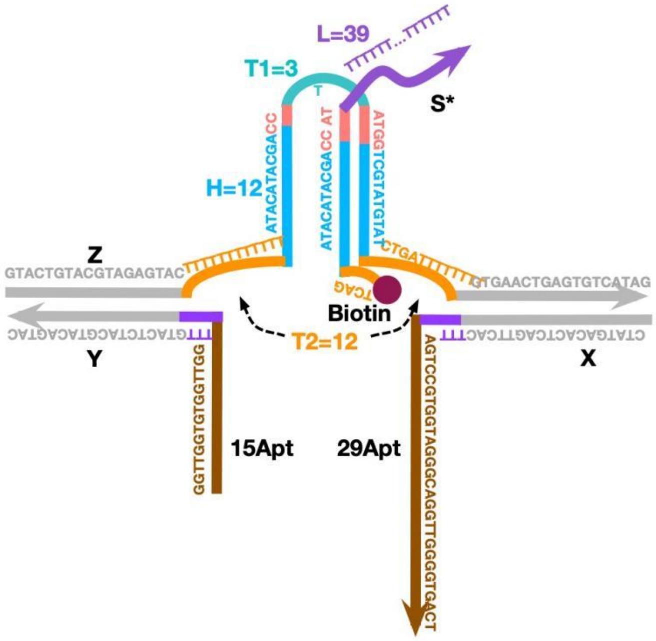

Supplementary Fig. 26 | Schematic diagram of DNA complex (XI).

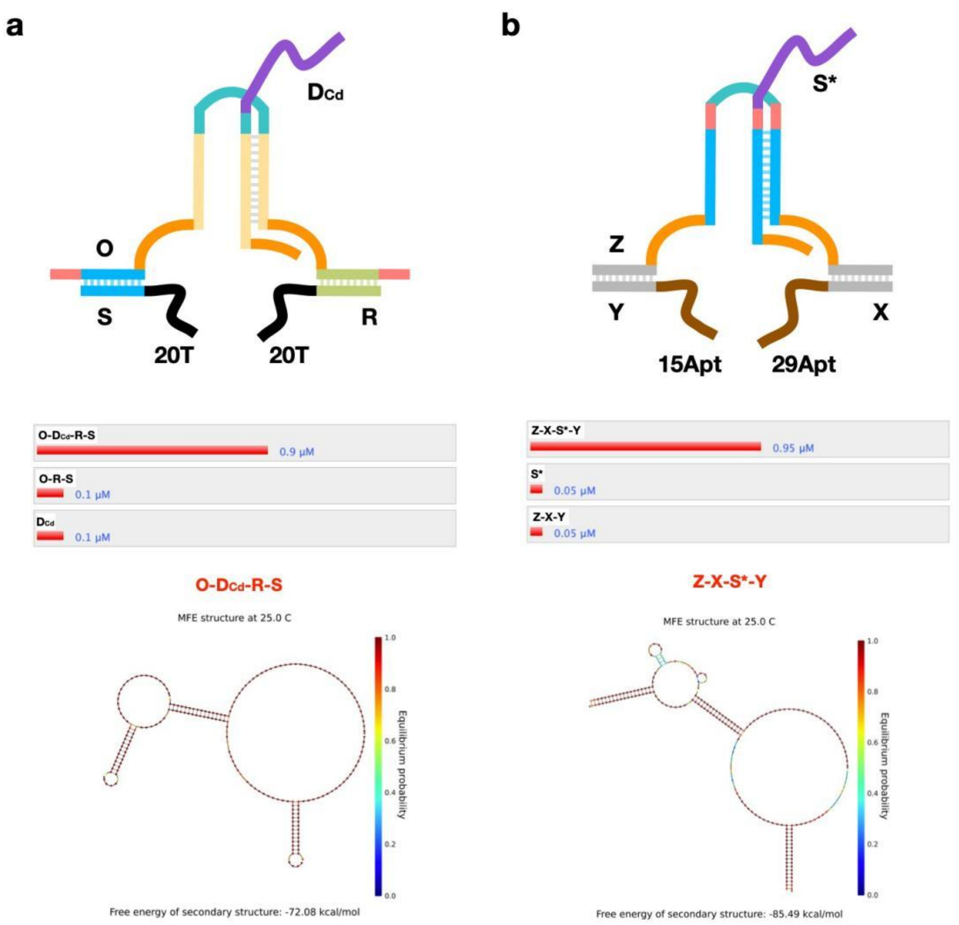

Supplementary Fig. 27 | NUPACK simulation results of the DNA complexes used in the switchable and cascade circuits. a, Schematic diagram and NUPACK simulation results of the assembly of complex (X) (at 25℃ and 1 μM concentration). **b**, Schematic diagram and NUPACK simulation results of the assembly of complex (XI) (at 25℃ and 1 μM concentration).

In order to realize cascaded control of CAST model, strands R* and S* are used as triggers, and a complete downstream translator structure is formed through strand displacement to complete downstream AST (Supplementary Fig. 28). Complex (X) was incubated with diaplacement strands R* and S* for 1h, and streptavidin was added for 1h. Fluorescence analysis showed that only when both the diaplacement strand and streptavidin were present, strand D_{Cd} release was generated and the fluorescence signal was significantly enhanced (Supplementary Fig. 28d and 28e).

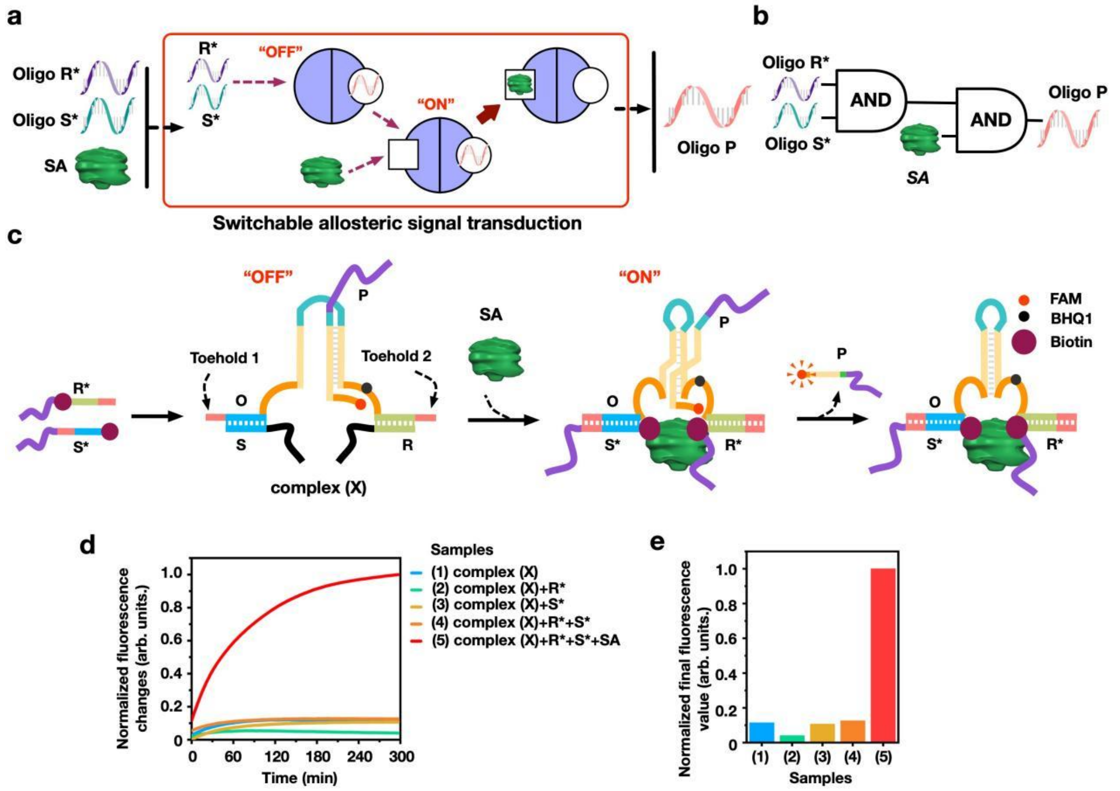

Supplementary Fig. 28 | Switchable AST circuit triggered by streptavidin. a,**b**, The illustrations and **c**, designs of switchable AST (using DNA complex (X), $OD_{Cd}RS$), respectively. **d**, Fluorescence results. [complex (X)] = 0.5 μM, R* = S* = 0.5 μM, [SA] = 1.5 μM. **e**, The final normalized fluorescence value of switchable AST.

S10. Designs and results of single-trigger-site CAST operations

S10.1 Designs and results of the single-trigger-site CAST operations triggered by thrombin

As concerned, it really needs to be addressed that whether the single binding site can trigger the CAST receptor. We first designed the single-trigger-site CAST receptor using one 29 nt aptamer to interact with thrombin protein (Supplementary Fig. 29). Specifically, the receptor was consisted of three DNA strands as C, D and E-29apt. In the gel results, a gradient increase of gel band intensities can be observed from lanes 2 to 9, when the thrombin concentrations was at 0.15 μM, 0.3 μM, 0.6 μM, 0.9 μM, 1.2 μM, 1.5 μM and 1.8 μM (Supplementary Fig. 30c). We also test the single-trigger-site based down-regulation module by introducing loop regulator T3 with the lengths varying from 3 to 30 nt. The fluorescence results of Fig. 4g are analyzed, and the results also show a down-regulation of the gradually decreasing density of the fluorescence signal as the length of the loop increases (Supplementary Fig. 30d). Meanwhile, in the gel results, the gradual decreasing band densities can be observed from lanes 7 to 11, with the increase of loop lengths (Supplementary Fig. 30e and 30f).

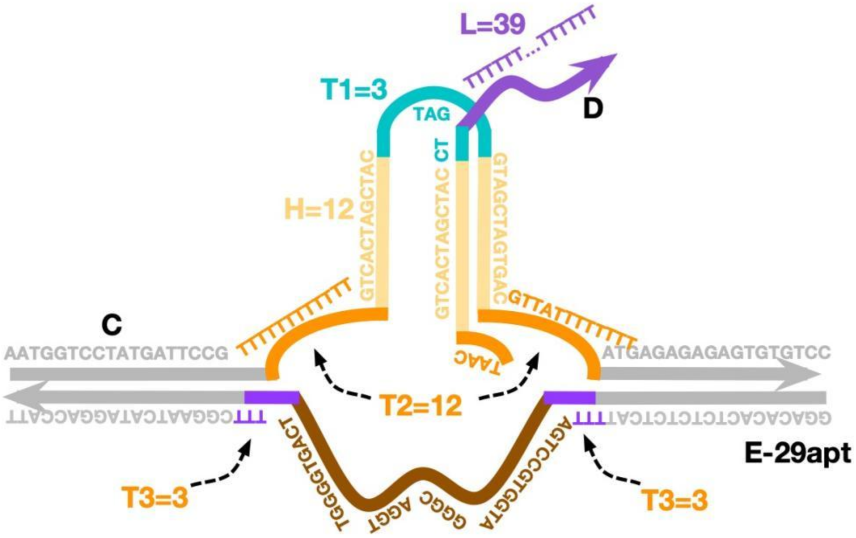

Supplementary Fig. 29 | Schematic diagram of the single-trigger-site CAST operations triggered by thrombin.

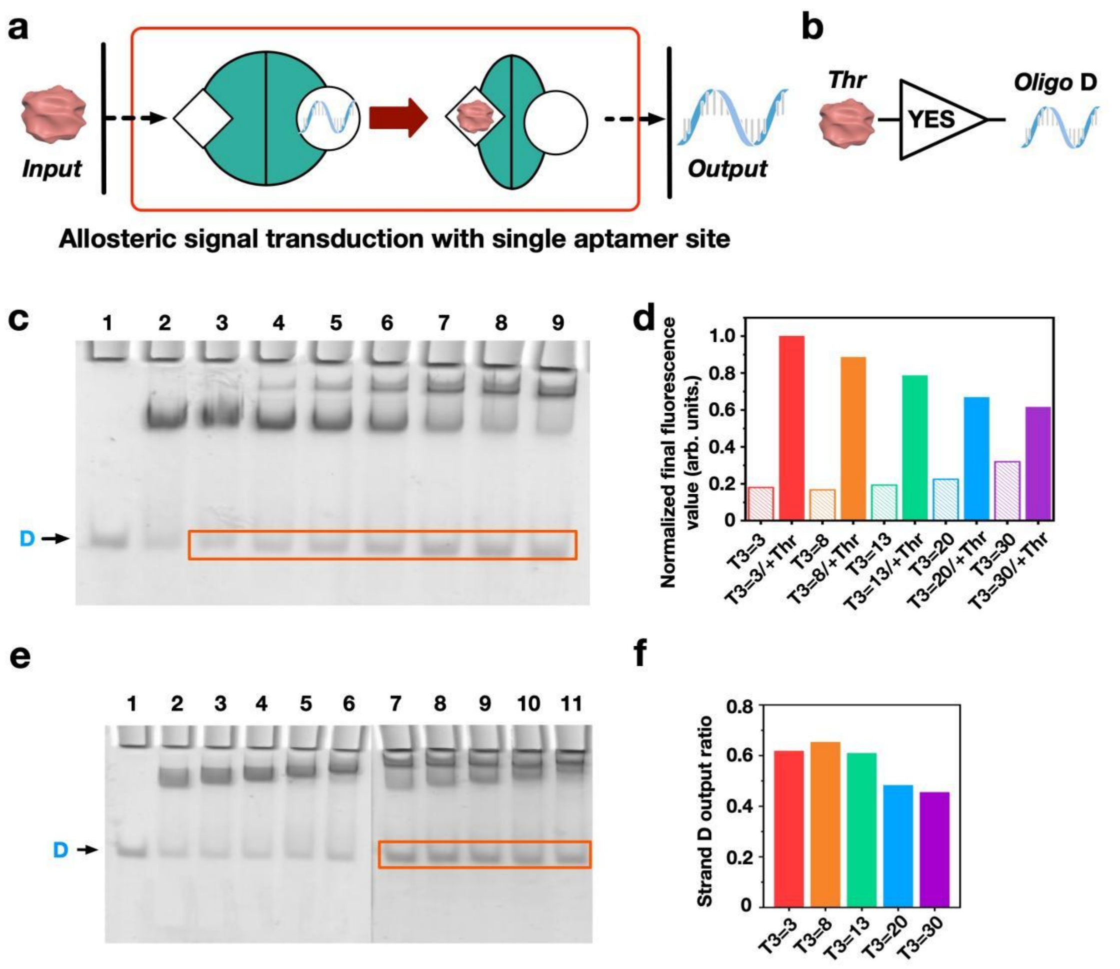

Supplementary Fig. 30 | Single-trigger-site CAST operations triggered by thrombin. **a**,**b**, Schematic and design illustration of single-trigger-site CAST triggered by thrombin. **c**, PAGE gel analysis of single-trigger-site CAST varying thrombin concentrations. **c**: [DNA complex] = 0.6 μM, [Thr] = 0, 0.15 μM, 0.3 μM, 0.6 μM, 0.9 μM, 1.2 μM, 1.5 μM and 1.8 μM. Lane 1 was AST output signal controls, and lane 2 was untriggered controls. **d**, Quantification of the final fluorescence values of the single-trigger-site CAST. **e**,**f**, Native PAGE (12% acrylamide) results (**e**), and statistical analysis (**f**), of single-trigger-site AST with different T3 lengths of 3, 8, 13, 20 and 30 nt for complex-T3 (1), (2), (3), (4) and (5). Lane 1: D, Lane 2: complex-T3 (1), Lane 3: complex-T3 (2), Lane 4: complex-T3 (3), Lane 5: complex-T3 (4), Lane 6: complex-T3 (5), Lane 7: complex-T3 (1) + Thr, Lane 8: complex-T3 (2) + Thr, Lane 9: complex-T3 (3) + Thr, Lane 10: complex-T3 (4) + Thr, Lane 11: complex-T3 (5) + Thr. [complex-T3] = 0.6 μM and [Thr] = 1.5 μM. Lanes 1-6 and lanes 7-11 are from two different PAGE gels for the same batch of experiments under exactly the same experimental conditions. Lanes 1 was AST output signal controls, and lanes 2-6 were untriggered controls.

S10.2 Designs and results of the single-trigger-site CAST operations triggered by PDGF-BB

We also tried to construct another single-trigger-site initiated CAST receptor by using protein PDGF-BB (Supplementary Fig. 31, Fig 32 a to c). At first, we used two kinds of PDGF-BB aptamers PDGF-35apt and PDGF-43apt to serve as the single-trigger-site to iniate the CAST receptor. However, the results of both of them were failed that the DNA D was not able to be carried in the initial CAST receptor. In the gel results in supplementary Fig. 32d, it is clear to see that a gel band of DNA D was left in lane 4 without hybridizing with the DNA complex. The possible reason may be that the secondary structures of the PDGF-BB aptamer spontaneously aggregate to form a complex self-hybridization structures, thus bringing the two ends of DNA C into a close proximity and make it difficult for DNA D to hybridize with the hairpin DNA C (Supplementary Fig. 32e and 32f).

Finally, we tried to construct the structure of CAST receptor with aptamers PDGF-43apt (Supplementary Fig. 32 g to i). Moreover, we tried to improve the PDGF-BB triggered single-site CAST receptor by optimizing the DNA sequences with H =11 nt and the hybridization lengths of C/D as 18 nt (Supplementary Fig. 33). However, the experimental results were still not satisfied. These experimental results indicated the constructions of the single-trigger-site CAST are different from the that of two-trigger-site CAST presented in original the manuscript. The reasons may lie in: 1) The separated design of aptamer trigger sites in the two-trigger-site CAST receptor is an important factor, which avoids the secondary structure to cause the initial aggregations; 2) The single-trigger-site CAST design itself is more susceptible to the influences of the secondary structure induced by the complex aptamer sequences. In addition, it is difficult to overcome even by carefully manual designs. Therefore, the universality of the single-trigger-site CAST is not as general as that of two-trigger-site CAST.

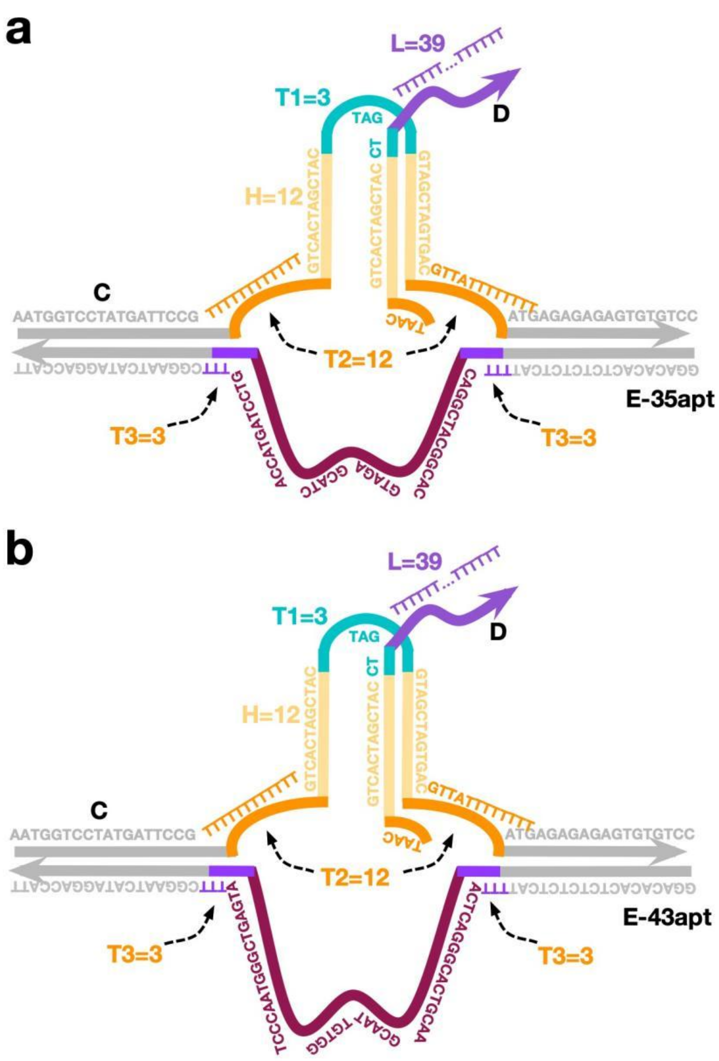

Supplementary Fig. 31 | Schematic diagram of the single-trigger-site CAST operations triggered by PDGF-BB (Two kinds of PDGF-BB). a,b, Schematic diagram of CDE-35apt and CDE-43apt, respectively.

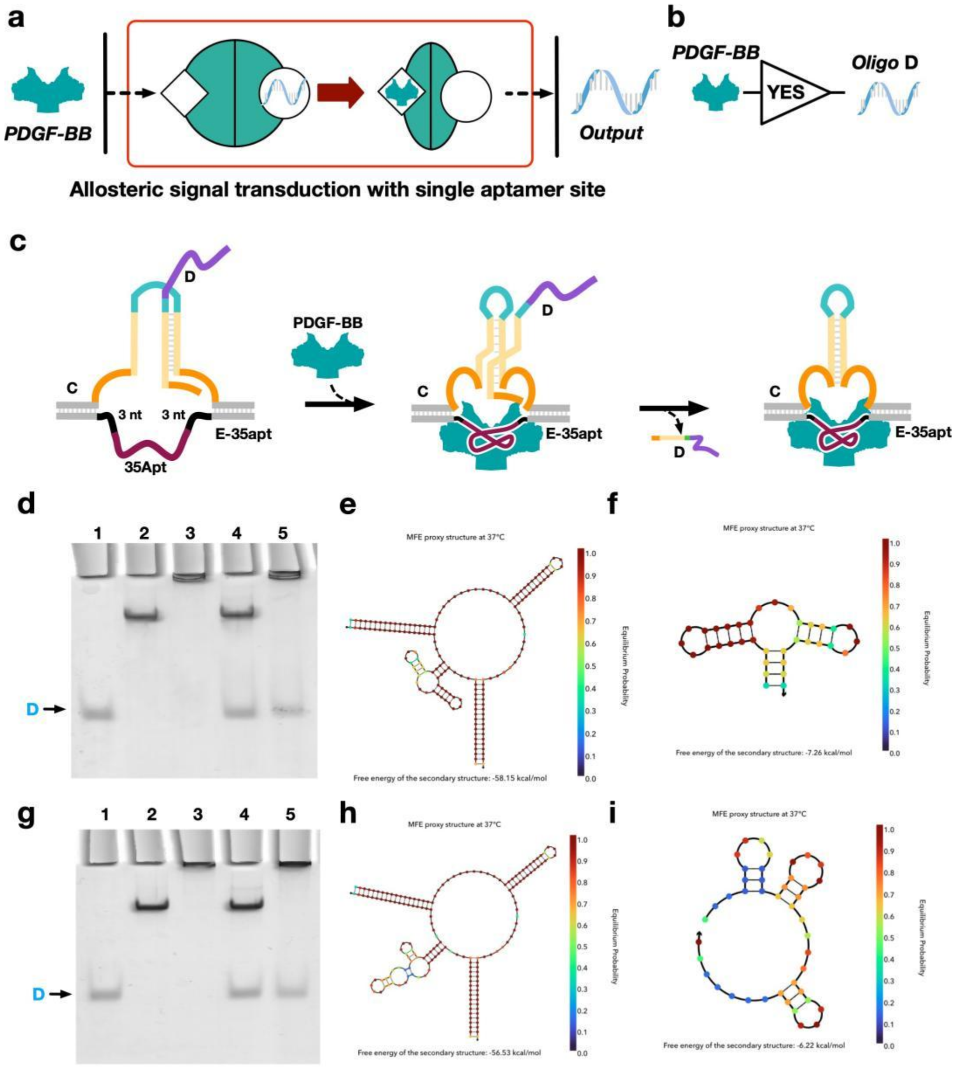

Supplementary Fig. 32 | Single-trigger-site CAST operations triggered by PDGF-BB. a,**b**,**c**, Schematic and design illustration of single-trigger-site CAST operations triggered by PDGF-BB. **d**, PAGE gel analysis of the single-trigger-site CAST operations triggered by PDGF-BB (aptamer PDGF-35apt). Lane 1: D; Lane 2: CE-35apt; Lane 3: CE-35apt + PDGF-BB; Lane 4: CDE-35apt; Lane 5: CDE-35apt + PDGF-BB. [DNA complex] = 0.6 μM, [PDGF-BB] = 1.5 μM. **e,** NUPACK simulation results of the assembly of strands C, and E-35apt (at 37°C and 1 μM concentration). **f**, NUPACK simulation results of the assembly of strand E-35apt (at 37°C and 1 μM concentration). **g**, PAGE gel analysis of the single-trigger-site CAST operations triggered by PDGF-BB (aptamer PDGF-43apt). Lane 1: D; Lane 2: CE-43apt; Lane 3: CE-43apt + PDGF-BB; Lane 4: CDE-43apt; Lane 5: CDE-43apt + PDGF-BB. [DNA complex] = 0.6 μM, [PDGF-BB] = 1.5 μM. **h**, NUPACK simulation results of the assembly of strands C, and E-43apt (at 37°C and 1 μM

concentration). **i**, NUPACK simulation results of the assembly of strand E-43apt (at 37°C and 1 μM concentration).

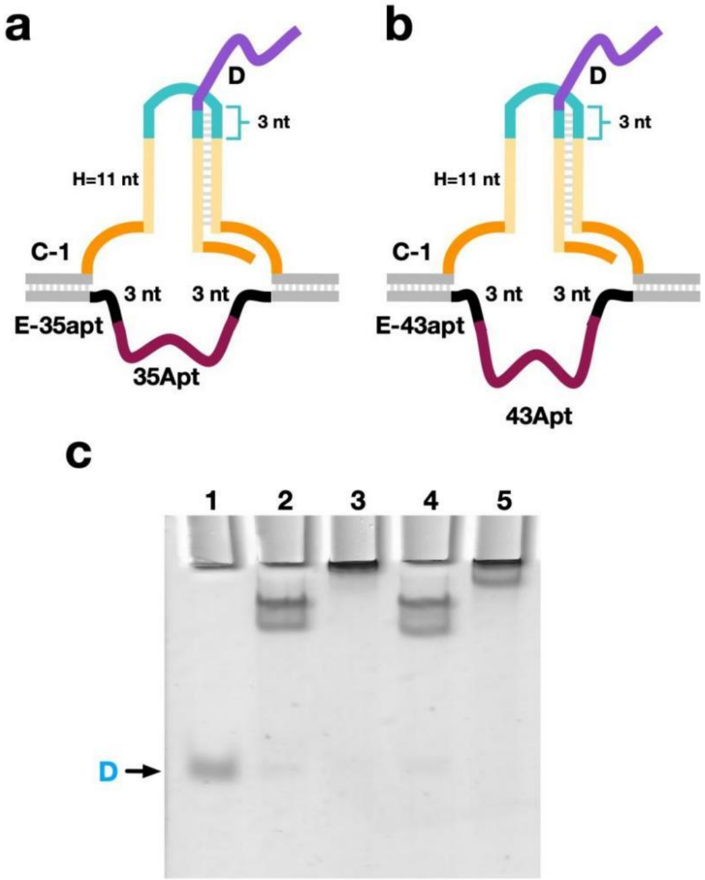

Supplementary Fig. 33 | Structural optimization of single-trigger-site CAST operations triggered by PDGF-BB. a,**b**, Structural optimization of single-trigger-site CAST operations triggered by PDGF-BB. **c**, PAGE gel analysis of the structural optimization of single-trigger-site CAST operations triggered by PDGF-BB. Lane 1: D; Lane 2: C-1DE-35apt; Lane 3: C-1DE-35apt + PDGF-BB; Lane 4: C-1DE-43apt; Lane 5: C-1DE-43apt + PDGF-BB. [DNA complex]= 0.6 μM, [PDGF-BB] = 1.5 μM.

S11. Using CAST strategy to regulate GFP gene expression

S11.1 Designs of the allosteric transducer to control GFP gene expression

We redesigned the strand D sequences with the GFP ASOs, called D_{GFP}, and so that the signal transduction induced allosteric release of DNA D_{GFP} could target at the GFP gene to regulate the gene expression in Hela cells. In the complex-G, the 3 'and 5' ends of DNA A and B are designed with thrombin aptamer sequences with the length of 29 nt and 15 nt, respectively, and the complementary domains of strand C are both 18 bp. The T1 domain of strand C is 3 nt, the T2 domains is 12 nt, and the H domain is 12 nt (Supplementary Fig. 34 and Table S10).

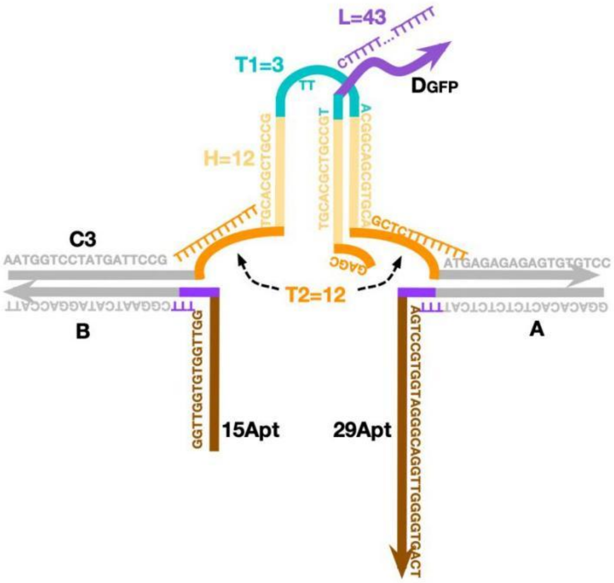

Supplementary Fig. 34 | Schematic diagram of DNA complex-G to control GFP gene expression.

We first tested whether complex-G could act in vitro. The results of PAGE experiment are shown in Supplementary Fig. 35. Upon adding thrombin concentration, the complex-G (C3D$_{GFP}$AB) combined with thrombin and formed C3AB + Thr complex, and the band moved upward. At the same time, strand D$_{GFP}$ is released from the basic structure.

After verifying that D$_{GFP}$ could be released, flow cytometry assay and confocal assay were used to verify whether D$_{GFP}$ could be released into Hela cells . We labeled single strand D$_{GFP}$ with Cy5.5 and Cy5 fluorophore and performed flow cytometry and confocal experiments, respectively. Flow cytometry (Supplementary Fig. 35c and 35d) and confocal experiment (Supplementary Fig. 36) showed that the fluorescence intensity in cells decreased significantly when thrombin was added to the complex-G compared with the condition without triggering. All these results confirmed that the nucleic acid strand released by the molecular translator could be taken up by Hela cells.

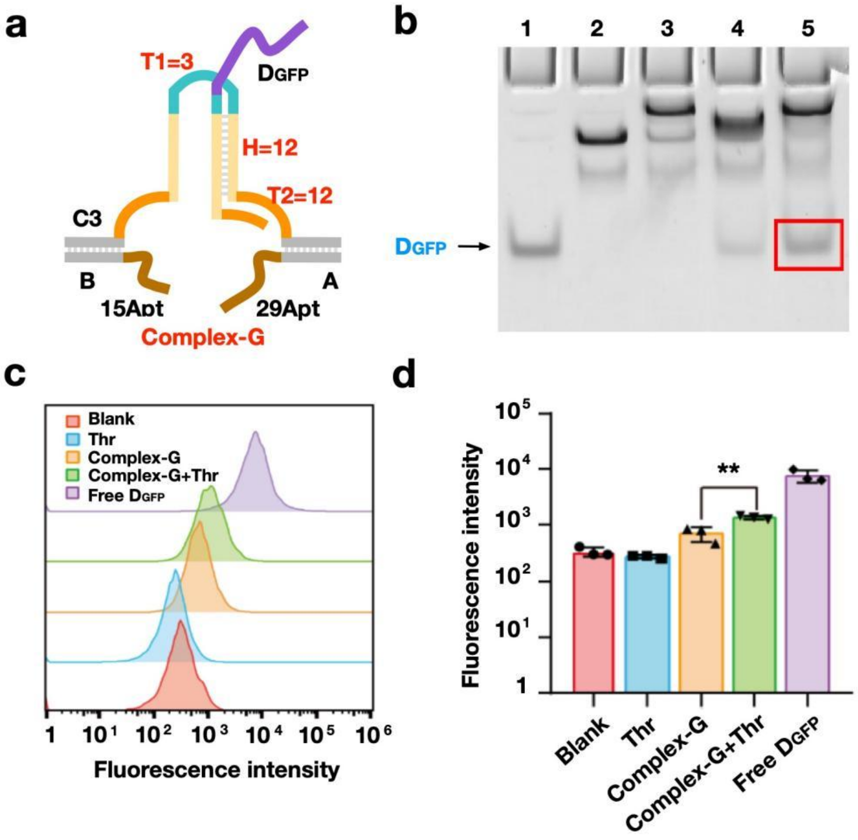

Supplementary Fig. 35 | PAGE and flow cytometry confirmed that AST regulates GFP gene expression. a, Design and **b**, PAGE results of the AST (using DNA Complex-G, C3D$_{GFP}$AB), respectively. Lane 1: D$_{GFP}$; Lane 2: C3AB; Lane 3: C3AB + Thr; Lane 4: Complex-G; Lane 5: Complex-G + Thr. **c**, Representative flow

cytometry image of Cy5.5-labeled nucleic acid strands being taken up by Hela cells (n = 3). **d**, Quantitative fluorescence intensity data from the flow cytometry results. Data collected in (**d**) were presented as the mean ± SD of n = 3 biologically independent experiments. Source data are provided as a Source Data file. Statistic analysis for (**d**) was performed using two-sided test (*p≦0.05, **p≦0.01, ***p≦0.001).

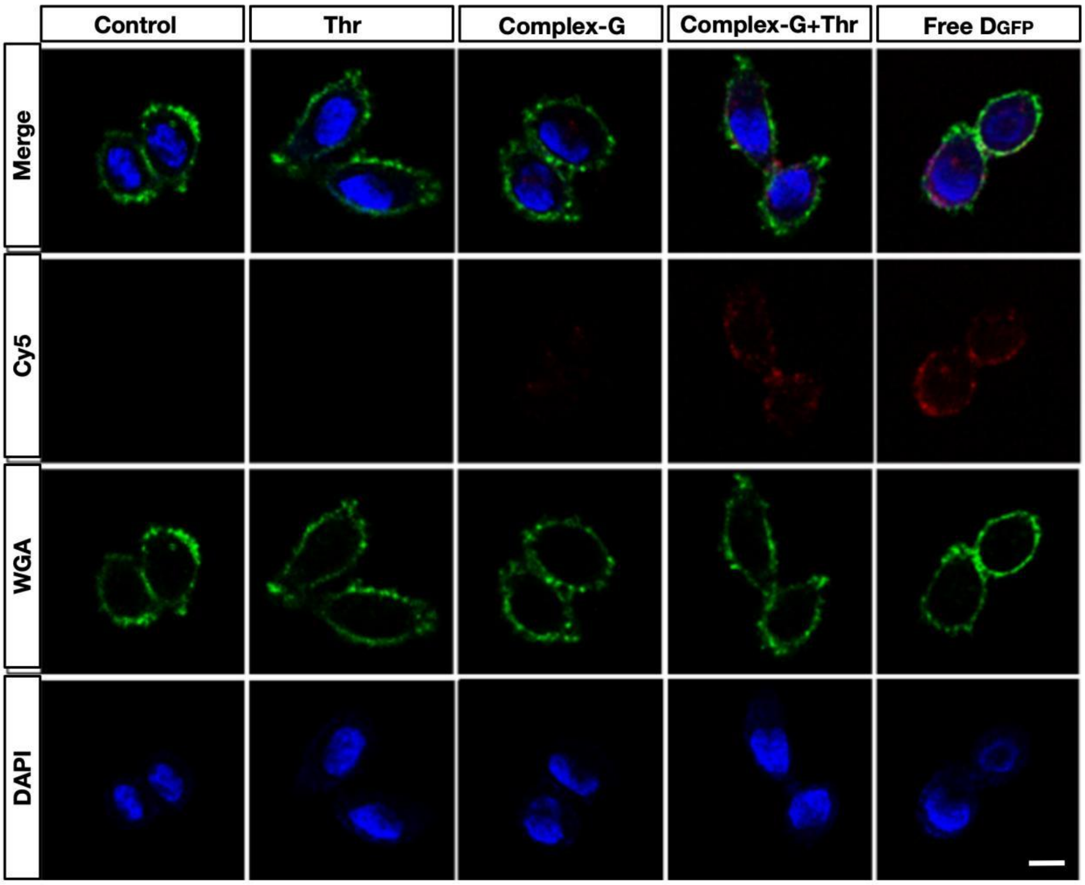

Supplementary Fig. 36 | Confocal experiments confirmed that the fluorescent Cy5-labeled nucleic acid strands could enter the interior of HeLa cells. Blue color is the nucleus; Green color is the cell membrane; The red color represents the Cy5-labeled strand D. Scale bars: 10 μm.

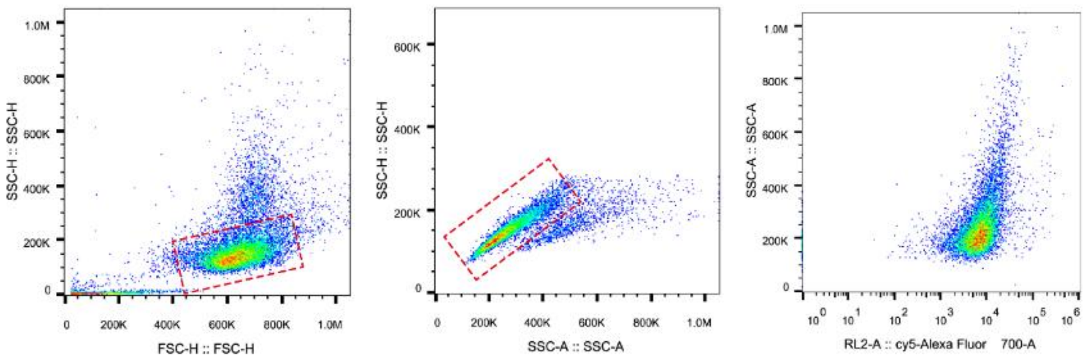

Supplementary Fig. 37 | Gating strategy for identification of the fluorescence of Hela cells

treated with Cy5-labeled nucleic acid strands. Hela cells from different treatment groups were gated· from·digested non-parenchymal single-cell suspensions following differential centrifugation.

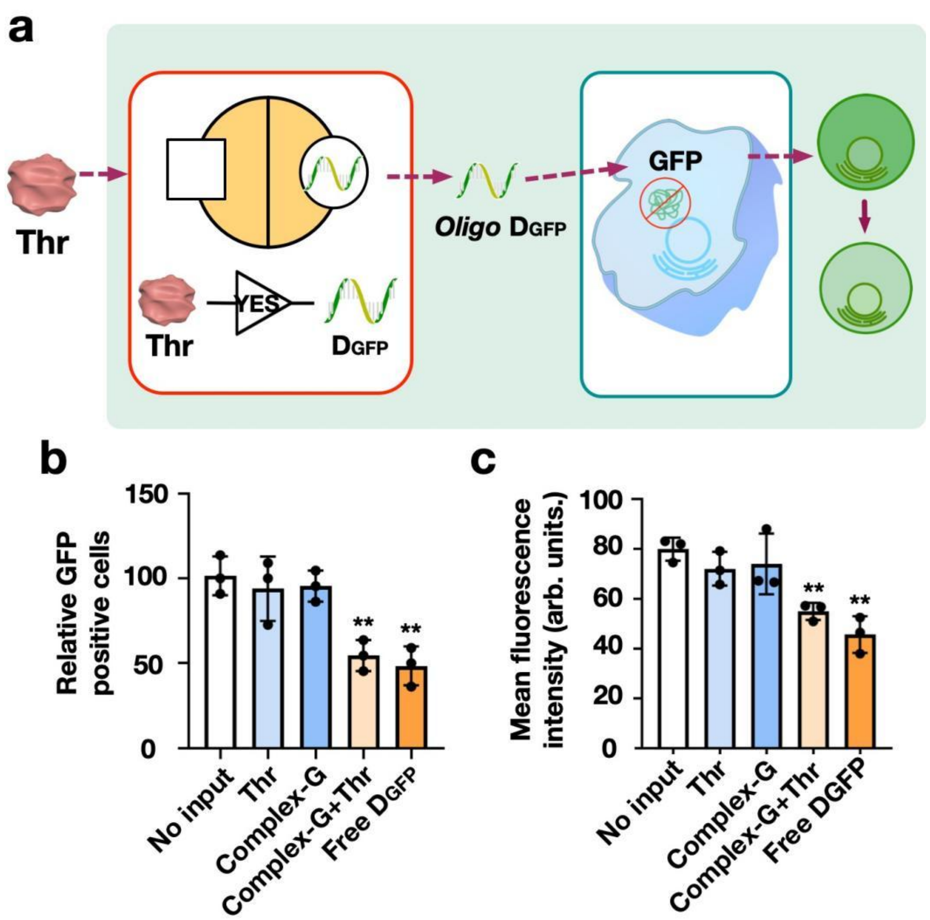

Supplementary Fig. 38 | Using AST to regulate GFP gene expression. a, Schematic illustration of the allosteric regulation of GFP gene expression via thrombin. **b**, Relative GFP positive cells results and **c**, mean fluorescence intensities of the allosteric regulations of GFP gene, respectively. Data collected in (**b**) and (**c**) were quantified using ImageJ software (Figure 5c) and are presented as mean ± s.d. for n = 3 biologically independent experiments. Source data are provided as a Source Data file. Statistic analysis for (**b**) and (**c**) was performed using two-sided test (*p≦0.05, **p≦0.01, ***p≦0.001).

S11.2 Cooperative regulation allosteric transducer to regulate GFP gene expression

In the study, we investigated to use using CAST strategy to regulate GFP gene expression. Based on the previous cooperative regulation mechanism, we designed six kinds of allosteric DNA complexes as shown in Supplementary Fig. 39 and Table S10. By varying the loop combinations of T1/T2, six kinds of DNA complexes were used in the experiments as: (i) C3-1D_{GFP}AB (T1 = 16 nt, T2 = 4 nt), (ii) C3-2D_{GFP}AB (T1 = 16 nt, T2 = 8 nt), (iii) C3-3D_{GFP}AB (T1 = 16 nt, T2 = 12 nt), (iv) C3-4D_{GFP}AB (T1 = 3 nt, T2 = 8 nt), (v) C3D_{GFP}AB (T1 = 3 nt, T2 = 12 nt) and (vi) C3-5D_{GFP}AB (T1 = 3 nt, T2 = 4 nt).

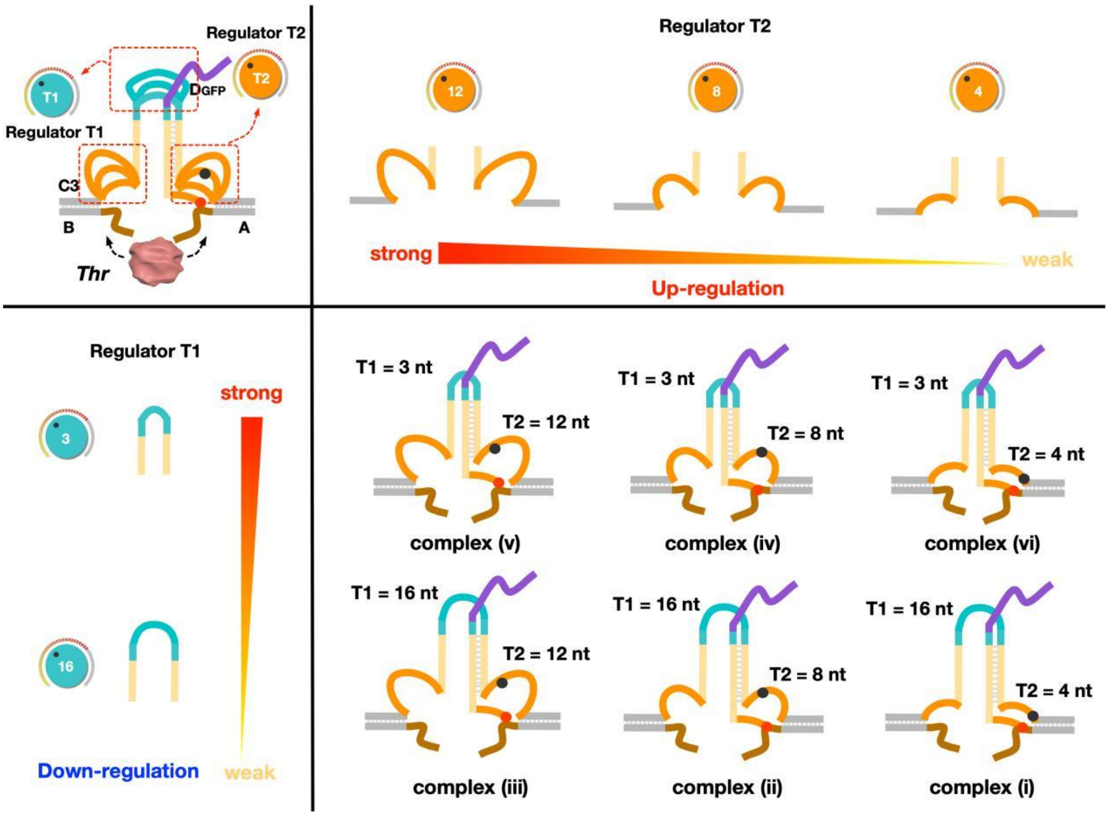

Supplementary Fig. 39 | Structural designs of all six kinds of cooperative regulations of DNA complexes to regulate GFP gene expression.

The PAGE experiments were used to verify the in vitro operations of CAST based gene regulation (Supplementary Fig. 40). In the gel results, it is clear to see that the releases of D_{GFP} can be found in the samples of thrombin triggered complexes (ii), (iii), (iv) and (v).

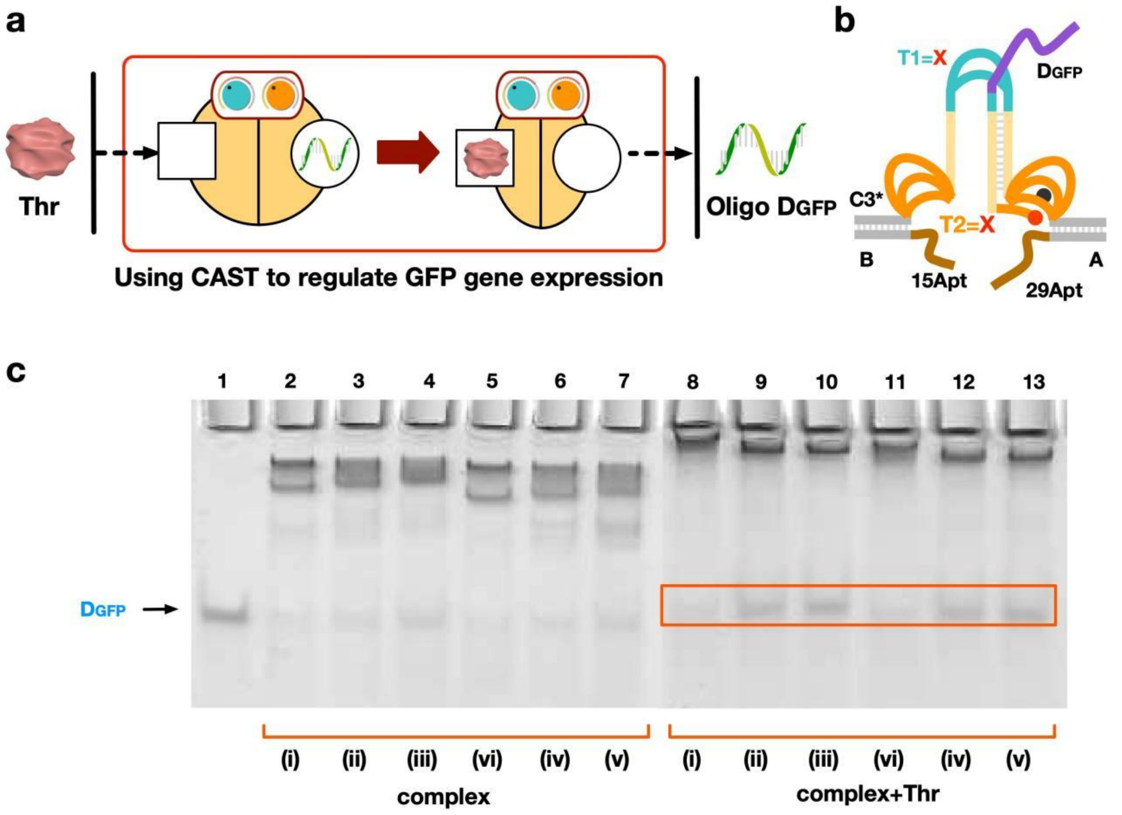

Supplementary Fig. 40 | Design and PAGE results of CAST regulation of GFP gene expression using CAST strategy. a, Schematic illustration and **b**, design of CAST regulations of GFP gene expression. **c**, PAGE results of CAST regulations of GFP gene expression. Lane 1: D_{GFP}; Lane 2: complex (i); Lane 3: complex (ii); Lane 4: complex (iii); Lane 5: complex (vi); Lane 6: complex (iv); Lane 7: complex (v); Lane 8: complex (i) + Thr; Lane 9: complex (ii) + Thr; Lane 10: complex (iii) + Thr; Lane 11: complex (vi) + Thr; Lane 12: complex (iv) + Thr; Lane 13: complex (v) + Thr. [complex] = 0.6 μM, [Thr] = 1.5 μM. Lanes 1-7 and lanes 8-13 are from two different PAGE gels for the same batch of experiments under exactly the same experimental conditions and the gels were processed in parallel. Lanes 1 was AST output signal controls, and lanes 2-7 were untriggered controls.

S12. Using CAST to regulate PLK1 gene expression

S12.1 Designs of the AST to regulate PLK1 gene expression

We developed a CAST based regulation of PLK1-specific ASOs to logically control PLK1 expression and influence the tumor cell proliferation. We firstly verified the released effect of the allosteric signal transduction of PLK1-specific ASOs (Table S11). As shown in Supplementary Fig. 41 and Fig. 42, PLK1-specific ASOs is initially captured in the DNA complex-P. In the complex, the 3 'and 5' ends of DNA A and B are designed with thrombin aptamer sequences with the length of 29 nt and 15 nt, respectively, and the complementary domains of strand C are both 18 bp. And the length of domain T1 of strand C is 3 nt, that of domain T2 is 12 nt, and that of domain H is 12 nt.

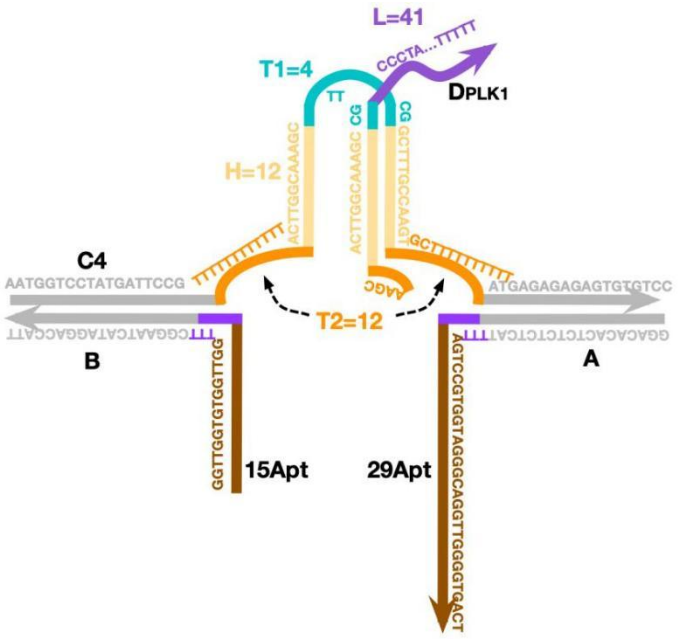

Supplementary Fig. 41 | Schematic diagram of DNA complex-P to control PLK1 gene expression.

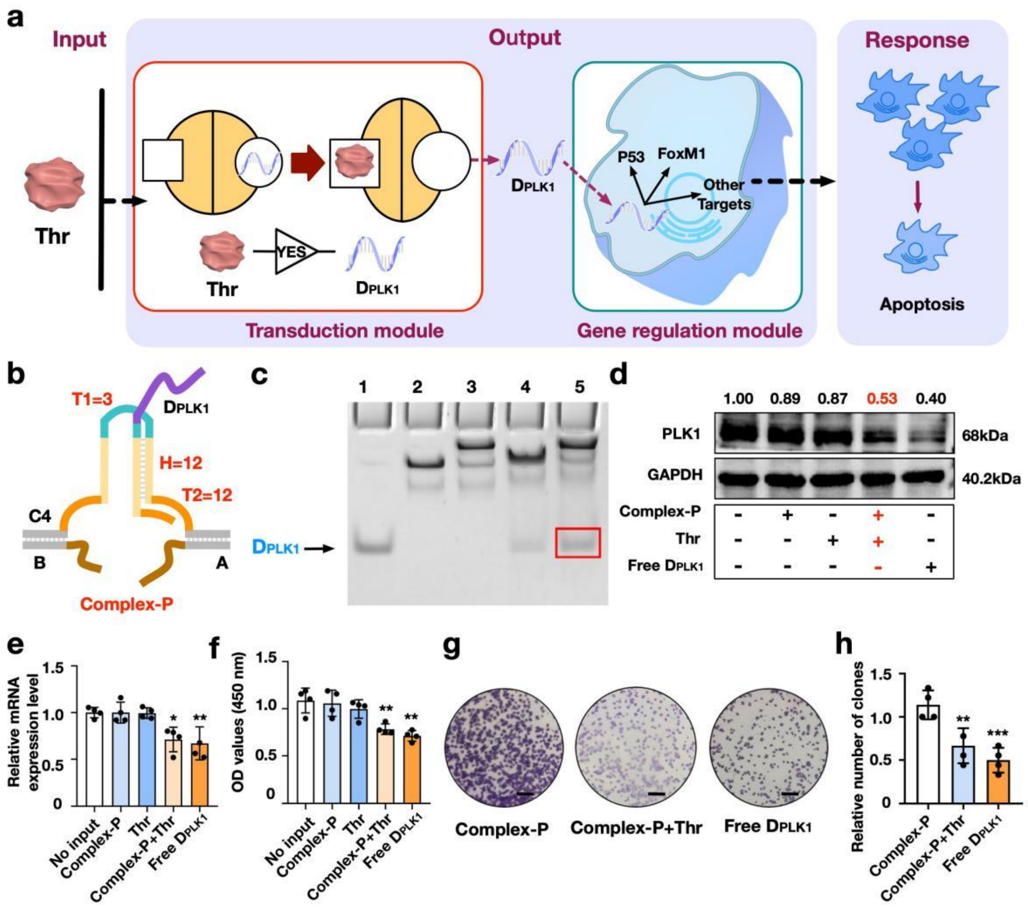

Supplementary Fig. 42 | Using allosteric "YES" gate to regulate PLK1 gene expression and influence cell proliferation. a, Schematics and **b**, design of the CAST "YES" gate regulations of PLK1 gene expression and tumor cell proliferations by using thrombin (using DNA Complex-P, C4D$_{PLK1}$AB), respectively. **c**, PAGE results of the "YES" gate regulations of PLK1 gene expression. Lane 1: D$_{PLK1}$; Lane 2: C4AB; Lane 3: C4AB + Thr; Lane 4: Complex-P; Lane 5: Complex-P + Thr. **d**, Western results of PLK1 gene expression. **e**, Relative mRNA expression results (examined by RT-qPCR). **f**, OD values results. **g**, The cell proliferation assay results. **h**, Statistical results of relative clones numbers in cell proliferation assay. **g**: Scale bars: 2 mm. Data collected in (**e**) and (**f**) are presented as mean ± s.d. for n = 4 biologically independent experiments. Data collected in (**h**) were quantified using ImageJ software and are presented as mean ± s.d. for n = 4 biologically independent experiments, source data provided. Statistic analysis for (**e**), (**f**) and (**h**) was performed using two-sided test (*p ≦ 0.05, **p ≦ 0.01, ***p ≦ 0.001). Source data are provided as a Source Data file.

S12.2 Two input logical operations to cooperatively regulates PLK1 gene based on CAST strategy

Based on the previous OR and AND logic operations, we developed the ASOs based CAST regulation of PLK1 gene expression. The DNA complex structural design is shown in Supplementary Fig. 43, Fig. 44 and Table S11. PAGE results of two-input logic operations indicated the efficient release of specific PLK1 ASOs in vitro as show in Supplementary Fig. 45.

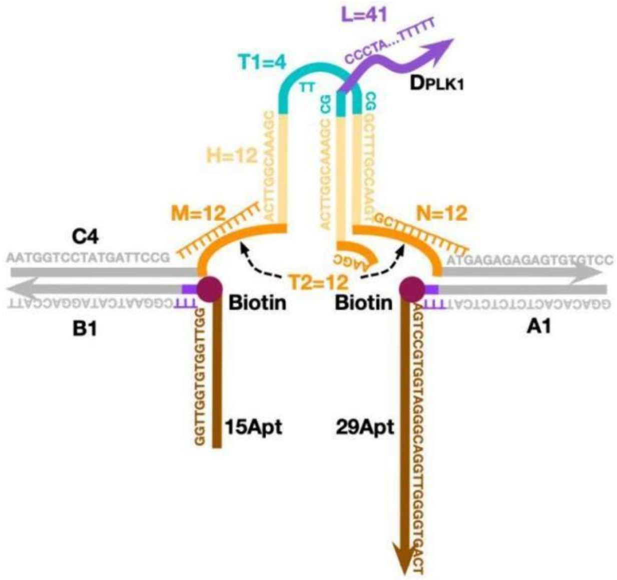

Supplementary Fig. 43 | Schematic diagram of DNA complex-OR to implement "OR" gate operations on PLK1 gene expression.

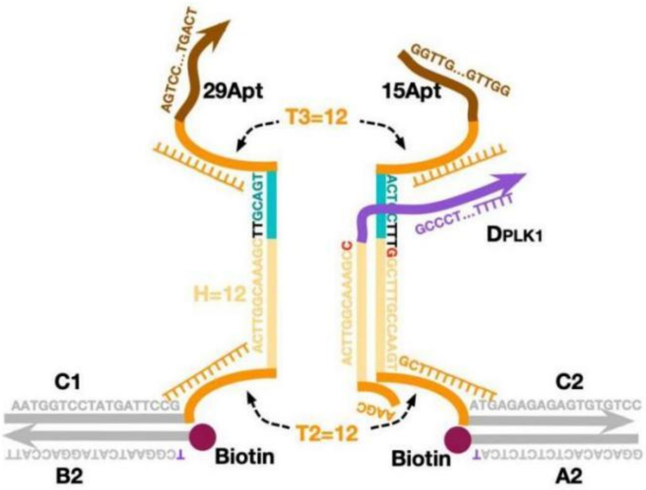

Supplementary Fig. 44 | Schematic diagram of DNA complex-AND to implement "AND" gate operations on PLK1 gene expression.

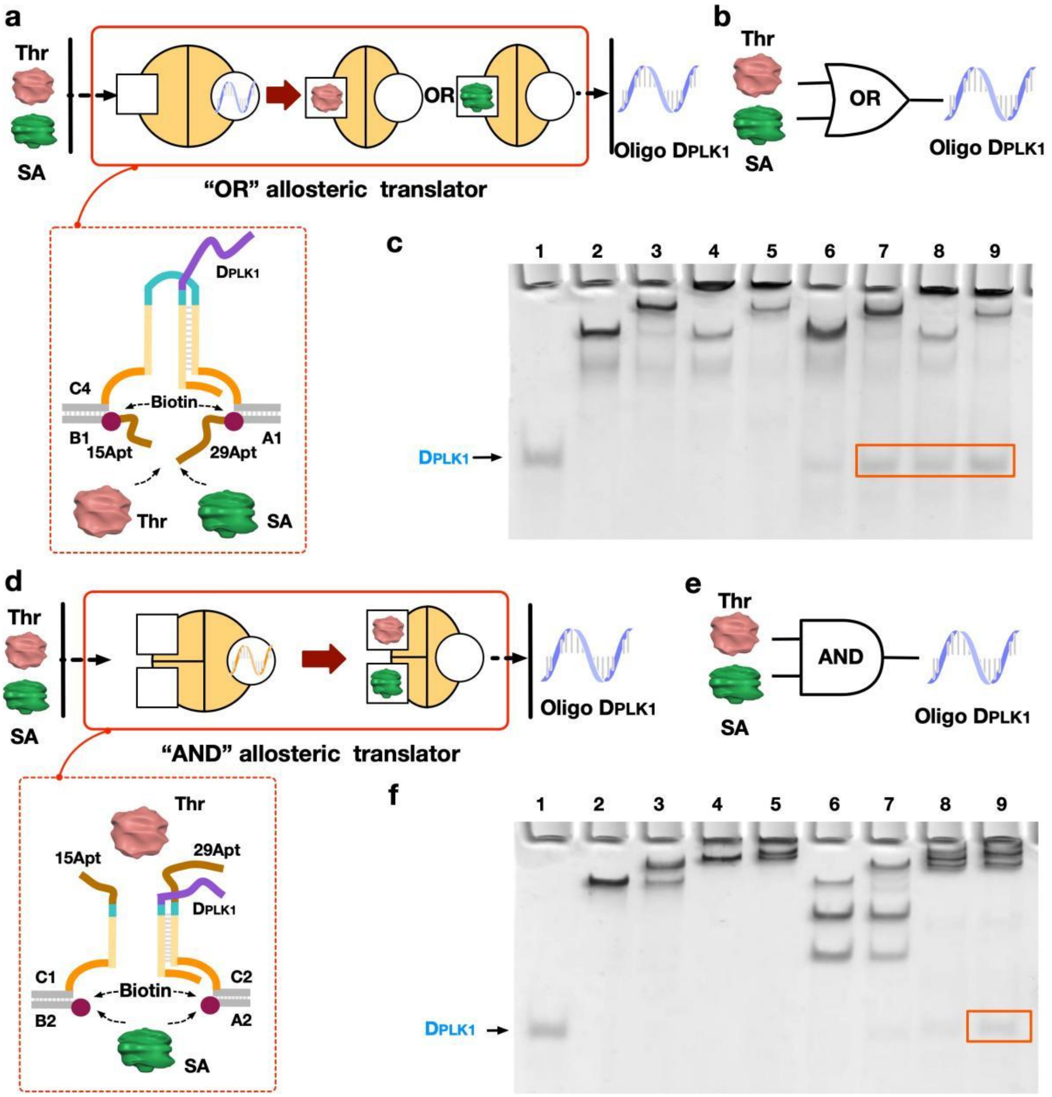

Supplementary Fig. 45 | PAGE results of two-input logic CAST operations to the release of specific PLK1 ASOs in vitro. a, Schematic illustration of the OR logic operations. **b**, Design and **c**, PAGE results of the OR logic operation, repectively. Lane 1: D_{PLK1}; Lane 2: C4A1B1; Lane 3: C4A1B1 + Thr; Lane 4: C4A1B1 + SA; Lane 5: C4A1B1 + Thr + SA; Lane 6: complex-OR; Lane 7: complex-OR + Thr; Lane 8: complex-OR + SA; Lane 9: complex-OR + Thr + SA. **d**,**e**, Design and **f**, PAGE results of the AND logic operation, respectively. Lane 1: D_{PLK1}; Lane 2: C1B2 + C2A2; Lane 3: C1B2 + C2A2 + Thr; Lane 4: C1B2 + C2A2 + SA; Lane 5: C1B2 + C2A2 + Thr + SA; Lane 6: complex-AND; Lane 7: C2A2 + SA; Lane 5: C1B2 + C2A2 + Thr + SA; Lane 6: complex-AND; Lane 7: complex-AND + Thr; Lane 8: complex-AND + SA; Lane 9: complex-AND + Thr + SA. [DNA complex] = 0.6 μM, [Thr] = 1.5 μM, [SA] = 1.8 μM. It is worth noting that in order to fit the next cell experiment, the reactions of DNA complex and protein were incubated at 37℃.

S13. Potential application scenarios of the CAST system

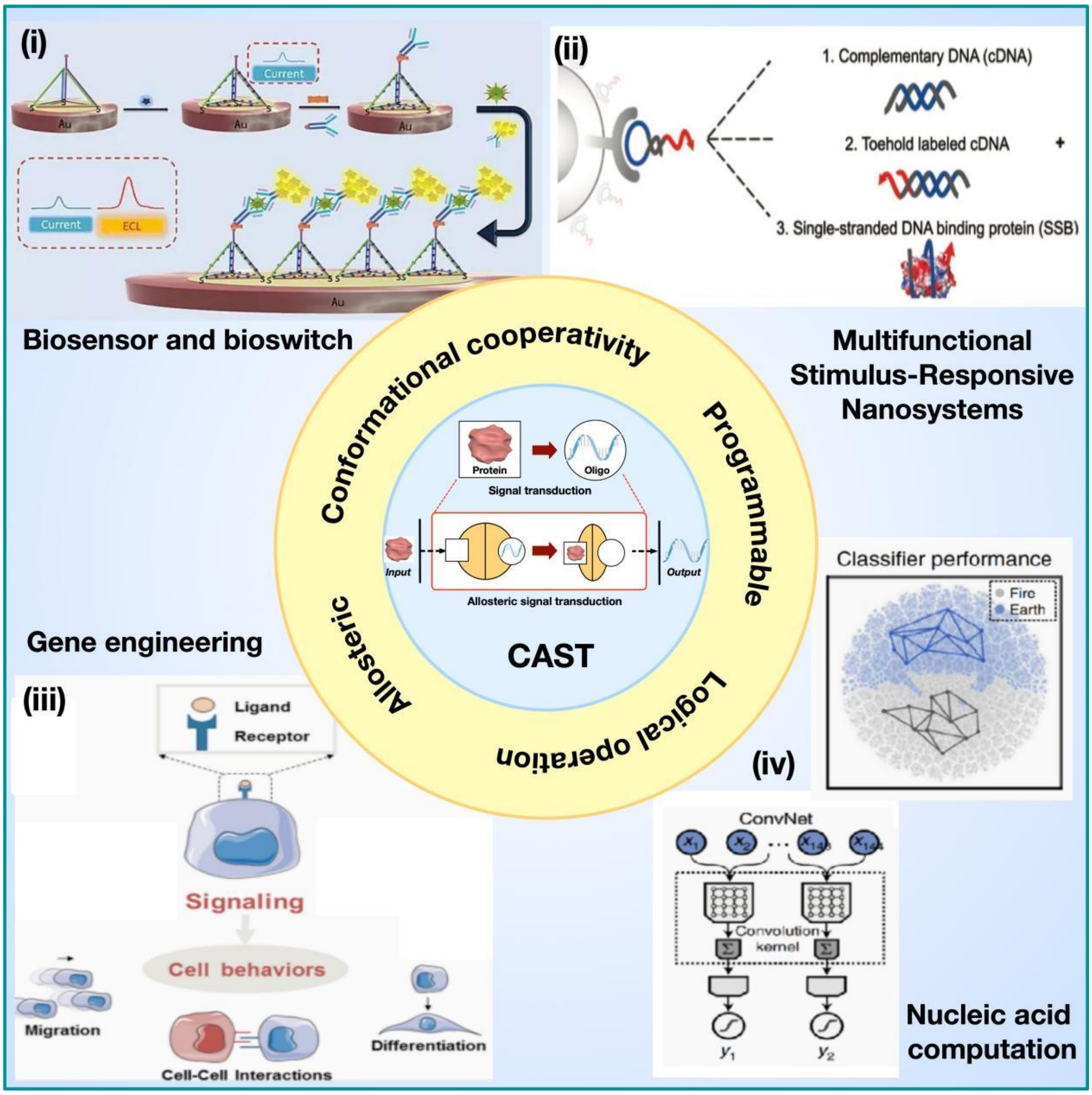

Supplementary Fig. 46 | Potential application scenarios of the CAST system. In the figures, four potential applications of the CAST system were shown, namely biosensor and bioswitch (i)[1], stimulating stimulus-responsive nanosystems (ii)[2], gene engineering (iii)[3] and nucleic acid computation (iv)[4]. All figures have been licensed accordingly.

S14. DNA devices triggered in intracellular and extracellular

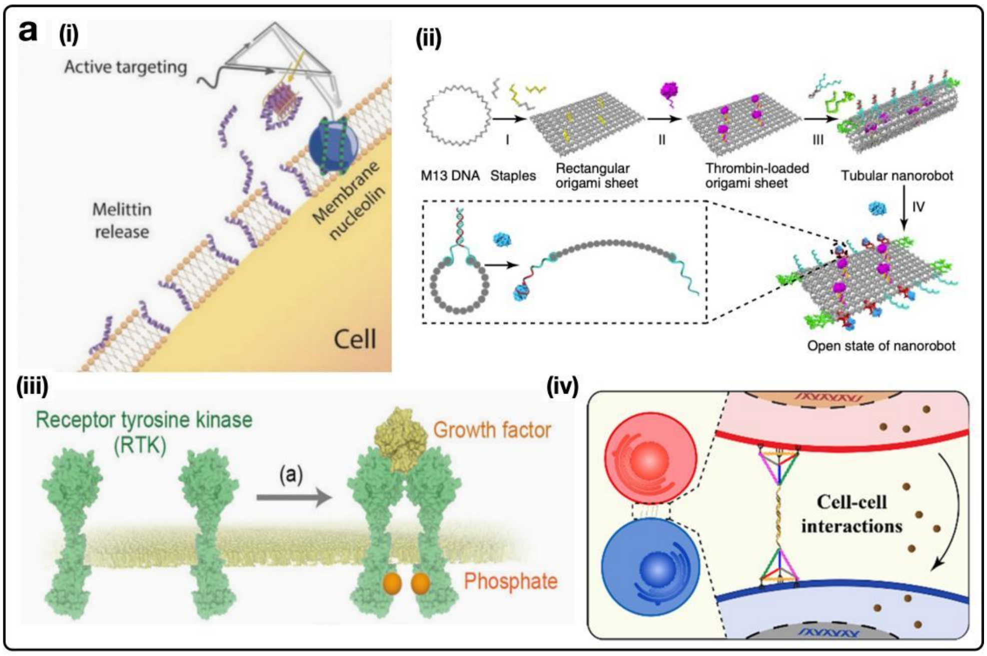

DNA devices triggered in extracellular context

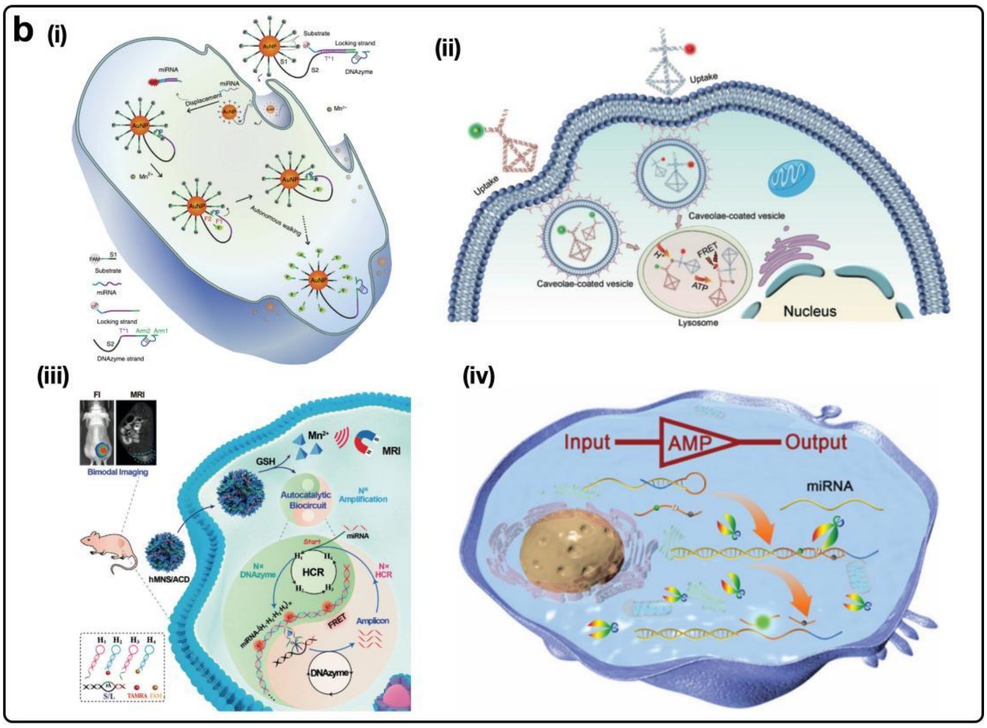

DNA devices triggered in intracellular context

Supplementary Fig. 47 | DNA devices triggered in intracellular and extracellular. **a**, Four scenarios for extracellular triggered DNA devices[5-8]. **b**, Four scenarios for intracellular triggered DNA devices[9-12]. All figures have been licensed accordingly.

Supplementary Tables

Table 1. Comparisons of the CAST and other molecule signal transduction methods.

Author	Triggers	Output	Trigger numbers	Applications	Regulation mode	References
Y. Liang et al.	Thrombin, Streptavidin	Oligonucleotide	1 or 2	ASO Gene regulation	Conformation cooperation, concentration	This work.
Q.L. Zhang et al.	ATP, Thrombin	Oligonucleotide	1	Nucleic acid computation	Concentration, conformation	13
W. Engelen et al.	Antibody	Oligonucleotide	2	Logic circuit	Concentration	14
C. Zhang et al.	Oligonucleotide	Oligonucleotide	1	Logic circuit, nanomachines	Conformation cooperation, concentration	15
S. Ranallo et al.	Antibody	Oligonucleotide	2	Modular DNA-based nanomachine	Concentration	16
S. Bracaglia et al.	Antibody	Transcribed RNA	2	Cell-free biosensor	Concentration, conformation	17
P. Li et al.	DNA	ATP	1	Transmembrane Transport	Concentration, light-controlled	18
L.A.P. Thompson et al.	DNA	ATP	1	Aptamer switches	Concentration, PH	19

Table 2. Comparisons of the CAST and the current ASO gene regulation methods.

Author	Modification and carrier tape	Regulation mode	Applications	Target	References
Y. Liang et al.	Ligand targeting	Conformation cooperation, concentration, logically control	Gene regulation,Cell apoptosis	GFP, PLK1	This work.
C. Xue et al.	Nanoparticles	Concentration	Cell apoptosis	PLK1	20
X.H. Wu et al.	Liposome carrying	Conformation, concentration	Gene Therapy	p53	21
G. A. O. Cremers et al.	DNA nanostructure	Concentration, DNA origami form	Cell surface receptor binding	PD1, EGFR, HER2	22
B. Cai et al.	Chemical modification	Concentration	Selection of DNA-encoded libraries to protein targets within and on living cells	Halotag-CBX7-ChD, SNAPtag-DOR	23
Q. Jiang et al.	DNA Origami	Concentration	Cell apoptosis	MCF-7 cell	24
Y. Zhang et al.	Nanoparticles	Concentration	Tumor therapy	miRNA-21	25
Z.H. Di et al.	Peptide nucleic acid	Concentration	Tumor therapy	caspase-3	26
D Hong et al.	Free uptake	Concentration	Tumor therapy	STAT3 RNA	27

Table 3. DNA systems for the extracellular triggering bioengineers.

Table 3. DNA systems for the extracellular triggering bioengineers.					
**Author**	**Name**	**Applications**	**Target**	**Trigger Pathway**	**References**
S.M. Traynor et al.	Bio-barcdoe assay	Cancer detection	Prostate specific antigen (PSA)	Human plasma/extracellular	28
B. Koos et al.	proxHCR	Protein Interactions and posttranslational modifications	E-cadherin and b-catenin in DLD1 cells	Cell surface /extracellular	29
L. Li et al.	Structure-switchable aptamer (SW-Apt)	Modulating aptamer specificity	PTK-7	Cell surface /extracellular	30
S.P. Li et al.	DNA nanorobot	Cancer therapeutic	Nucleolin	Cell surface /extracellular	31
Y.Y. Sun et al.	DNA- origami-based pMHC multimers	Antigen-specific CD8+ T cell detection	T cell	Cell surface /extracellular	32
T. Shibata et al.	Protein-driven RNAnanostructured devices	Regulate mammalian cell fate	L7Ae	Extracellular	33
Y.J. Wang et al.	Xeno-nucleic-acid-modifiedclassic DNAzyme	Silences gene expression	RNA substrate	Extracellular	34
M.S. Xiao et al.	DNA reaction circuits	Programming multiple cell-cell interactions	Multiple cell	Extracellular	35
J. Li et al.	3D amphiphilic pyramidal DNA	Cellular interactions	Multiple cell	Extracellular	36

Table 4. DNA based gene regulations with outside cellular triggering.

Table 4. DNA based gene regulations with outside cellular triggering.					
**Author**	**Name**	**Applications**	**Target**	**Trigger Pathway**	**References**
K. Jiao et al.	Topologically Ordered DNA	RNA transcription	T7 promoter	Extracellular	37
Y.J. Wang et al.	Xeno-nucleic-acid-modified classic DNAzyme	Silences gene expression	RNA substrate	Extracellular and intracellular	34
T. Shibata et al.	Protein-driven RNA nanostructured devices	Regulate mammalian cell fate	L7Ae	Extracellular	33
D. Han et al.	ssDNA probe (Apt-S-T)	Cell isolation	Sgc8c-S-T1,TCO1-S-T2,Sgc4f-S-T3	Extracellular	38
K. Zagorovsky et al.	MNAzyme	Disease diagnosis	RNA substrate	Extracellular	39
S. Angerani et al.	Ligands functionalized with peptide nucleic acids	Responsive membrane dimer protein	Carbonic anhydrases	Cell surface /extracellular	40
Ishaqat A et al.	CpG ODNs	Immunostimulation	TLR9	Extracellular	41

Table S1. DNA sequence of the basic allosteric signal transduction system

Name	Sequence	Modification
C	AATGGTCCTATGATTCCGTTTTTTTTTTTTGTC ACTAGCTACTAGGTAGCTAGTGACGTTATTTT TTTTATGAGAGAGAGTGTGTCC	T-BHQ1 (Red base modification)
D	TAACGTCACTAGCTACCTTTTTTTTTTTTTTTT TTTTTTTTTTTTTTTTTTTTTTTT	5'-FAM
A	GGACACACTCTCTCTCATTTTAGTCCGTGGTA GGGCAGGTTGGGGTGACT	
B	GGTTGGTGTGGTTGGTTTCGGAATCATAGGAC CATT	

Table S2. DNA sequence of change the length of regulator T2

Name	Sequence	Modification
C1*	AATGGTCCTATGATTCCGTTTTGTCACTAGCT ATAGTAGCTAGTGACGTTAATGAGAGAGAGT GTGTCC	T-BHQ1
C2*	AATGGTCCTATGATTCCGTTTTTTTTGTCACTA GCTATAGTAGCTAGTGACGTTATTTTATGAGA GAGAGTGTGTCC	T-BHQ1
C3*	AATGGTCCTATGATTCCGTTTTTTTTTTTTGTC ACTAGCTATAGTAGCTAGTGACGTTATTTTTT TTATGAGAGAGAGTGTGTCC	T-BHQ1
C4*	AATGGTCCTATGATTCCGTTTTTTTTTTTTTTT TGTCACTAGCTATAGTAGCTAGTGACGTTATT TTTTTTTTTATGAGAGAGAGTGTGTCC	T-BHQ1
D1*	TAACGTCACTAGCTACTTTTTTTTTTTTTTTTT TTTTTTTTTTTTTTTTTTTTTTT	5'-FAM
A	GGACACACTCTCTCTCATTTTAGTCCGTGGTA GGGCAGGTTGGGGTGACT	
B	GGTTGGTGTGGTTGGTTTCGGAATCATAGGAC CATT	
A2	GGACACACTCTCTCTCAT	3'-biotin

B2	TCGGAATCATAGGACCATT	5'-biotin

Table S3. DNA sequence of varying the length of hairpin H

Name	Sequence	Modification
C5*	AATGGTCCTATGATTCCGTTTTTTTTTTTTGTC ACTAGCTTAGAGCTAGTGACGTTATTTTTTTT ATGAGAGAGAGTGTGTCC	T-BHQ1
C6*	AATGGTCCTATGATTCCGTTTTTTTTTTTTGTC ACTAGCTATAGTAGCTAGTGACGTTATTTTTT TTATGAGAGAGAGTGTGTCC	T-BHQ1
C7*	AATGGTCCTATGATTCCGTTTTTTTTTTTTGTC ACTAGCTACTAGGTAGCTAGTGACGTTATTTT TTTTATGAGAGAGAGTGTGTCC	T-BHQ1
C8*	AATGGTCCTATGATTCCGTTTTTTTTTTTTGTC ACTAGCTAGATAGTCTAGCTAGTGACGTTATT TTTTTTATGAGAGAGAGTGTGTCC	T-BHQ1
D2*	TAACGTCACTAGCTCTTTTTTTTTTTTTTTTTT TTTTTTTTTTTTTTTTTTTTT	5'-FAM
D3*	TAACGTCACTAGCTACTTTTTTTTTTTTTTTTT TTTTTTTTTTTTTTTTTTTTTTT	5'-FAM
D4*	TAACGTCACTAGCTACCTTTTTTTTTTTTTTTT TTTTTTTTTTTTTTTTTTTTTTTT	5'-FAM
D5*	TAACGTCACTAGCTAGACTTTTTTTTTTTTTTT TTTTTTTTTTTTTTTTTTTTTTTTT	5'-FAM
A	GGACACACTCTCTCTCATTTTAGTCCGTGGTA GGGCAGGTTGGGGTGACT	
B	GGTTGGTGTGGTTGGTTTCGGAATCATAGGAC CATT	
A2	GGACACACTCTCTCTCAT	3'-biotin
B2	TCGGAATCATAGGACCATT	5'-biotin

Table S4. DNA sequence of the AST triggered by streptavidin

Name	Sequence	Modification
C6*	AATGGTCCTATGATTCCGTTTTTTTTTTTTGTC ACTAGCTATAGTAGCTAGTGACGTTATTTTTT TTATGAGAGAGAGTGTGTCC	T-BHQ1
D3*	TAACGTCACTAGCTACTTTTTTTTTTTTTTTTT TTTTTTTTTTTTTTTTTTTTTTT	5'-FAM
A2	GGACACACTCTCTCTCAT	3'-biotin
B2	TCGGAATCATAGGACCATT	5'-biotin

Table S5. DNA sequence of the AST triggered by PDGF-BB

Name	Sequence	Modification
C	AATGGTCCTATGATTCCGTTTTTTTTTTTTGTC ACTAGCTACTAGGTAGCTAGTGACGTTATTTT TTTTATGAGAGAGAGTGTGTCC	T-BHQ1
D	TAACGTCACTAGCTACCTTTTTTTTTTTTTTTT TTTTTTTTTTTTTTTTTTTTTTTT	5'-FAM
A3	GGACACACTCTCTCTCATTTTCAGGCTACGGC ACGTAGAGCATCACCATGATCCTG	
B3	CAGGCTACGGCACGTAGAGCATCACCATGAT CCTGTTTCGGAATCATAGGACCATT	

Table S6. DNA sequences of CAST using regulators T1 and T2

Name	Sequence	Modification
C9*	AATGGTCCTATGATTCCGTTTTTTTTTTTTGT CACTAGCTACTAGGTAGCTAGTGACGTTATT TTTTTTATGAGAGAGAGTGTGTCC	T-BHQ1
C10*	AATGGTCCTATGATTCCGTTTTTTTTGTCACT AGCTACTAGGTAGCTAGTGACGTTATTTTAT GAGAGAGAGTGTGTCC	T-BHQ1
C11*	AATGGTCCTATGATTCCGTTTTGTCACTAGC TACTAGGTAGCTAGTGACGTTAATGAGAGA GAGTGTGTCC	T-BHQ1
C12*	AATGGTCCTATGATTCCGTTTTTTTTTTTTGT CACTAGCTACTTTTTTTTTTTTTTTAGGTAGCT AGTGACGTTATTTTTTTTATGAGAGAGAGTG TGTCC	T-BHQ1
C13*	AATGGTCCTATGATTCCGTTTTTTTTGTCACT AGCTACTTTTTTTTTTTTTTTAGGTAGCTAGTG ACGTTATTTTATGAGAGAGAGTGTGTCC	T-BHQ1
C14*	AATGGTCCTATGATTCCGTTTTGTCACTAGC TACTTTTTTTTTTTTTTTAGGTAGCTAGTGACG TTAATGAGAGAGAGTGTGTCC	T-BHQ1
C15*	AATGGTCCTATGATTCCGTTTTTTTTTTTTGT CACTAGCTACTTTTTTTTTTTTTTTTTTTTTTT AGGTAGCTAGTGACGTTATTTTTTTTATGAG AGAGAGTGTGTCC	T-BHQ1
C16*	AATGGTCCTATGATTCCGTTTTTTTTGTCACT AGCTACTTTTTTTTTTTTTTTTTTTTTTTTAGGT AGCTAGTGACGTTATTTTATGAGAGAGAGTG TGTCC	T-BHQ1
C17*	AATGGTCCTATGATTCCGTTTTGTCACTAGC TACTTTTTTTTTTTTTTTTTTTTTTTTAGGTAGC TAGTGACGTTAATGAGAGAGAGTGTGTCC	T-BHQ1
D	TAACGTCACTAGCTACCTTTTTTTTTTTTTTT	5'-FAM

	TTTTTTTTTTTTTTTTTTTTTTTTT	
A	GGACACACTCTCTCTCATTTAGTCCGTGGT AGGGCAGGTTGGGGTGACT	
B	GGTTGGTGTGGTTGGTTTCGGAATCATAGGA CCATT	

Table S7. OR logic operations sequences

Name	Sequence	Modification
C	AATGGTCCTATGATTCCGTTTTTTTTTTTTGT CACTAGCTACTAGGTAGCTAGTGACGTTATT TTTTTTATGAGAGAGAGTGTGTCC	T-BHQ1
D	TAACGTCACTAGCTACCTTTTTTTTTTTTTTT TTTTTTTTTTTTTTTTTTTTTTTTT	5'-FAM
A1	GGACACACTCTCTCTCATTTTAGTCCGTGGT AGGGCAGGTTGGGGTGACT	T-biotin (Red base modification)
B1	GGTTGGTGTGGTTGGTTTCGGAATCATAGGA CCATT	T-biotin

Table S8. AND logic operations sequences

Name	Sequence	Modification
C1-1	AATGGTCCTATGATTCCGTTTTTTTTTTTTAC TTGGCAAAGCTTTTTTTTTTTTAGTCCGTGGT AGGGCAGGTTGGGGTGACT	
C2-1	GGTTGGTGTGGTTGGTTTTTTTTTTTTCGGCT TTGCCAAGTGCTTTTTTTTTTATGAGAGAGA GTGTGTCC	
C2-2	GGTTGGTGTGGTTGGTTTTTTTTTTTTTGGCT TTGCCAAGTGCTTTTTTTTTTATGAGAGAGA GTGTGTCC	
C1	AATGGTCCTATGATTCCGTTTTTTTTTTTTAC TTGGCAAAGCTTGCAGTTTTTTTTTTTTTAGT	

	CCGTGGTAGGGCAGGTTGGGGTGACT	
C2-3	GGTTGGTGTGGTTGGTTTTTTTTTTTTACTGC TTCGGCTTTGCCAAGTGCTTTTTTTTTTTATGA GAGAGAGTGTGTCC	
C2	GGTTGGTGTGGTTGGTTTTTTTTTTTTACTGC TTTGGCTTTGCCAAGTGCTTTTTTTTTTTATGA GAGAGAGTGTGTCC	
D_{AND}	AAGCACTTGGCAAAGCCGCCCTATTCATTCT TCTTGATCCGGTTTTTTTTTTTTTTTTT	5’-FAM
A2	GGACACACTCTCTCTCAT	3’-biotin
B2	TCGGAATCATAGGACCATT	5’-biotin

Table S9. Cascade circuit sequences

Name	Sequence	Modification
O	ATGGTCGTATGTATCTGTTTTTTTTTTTTGTC ACTAGCTATAGTAGCTAGTGACGTTATTTTT TTTGAAGACAGTCAGATCGA	T-BHQ1
D_{Cd}	TAACGTCACTAGCTACTTTTTTTTTTTTTTTT TTTTTTTTTTTTTTTTTTTTTTTTT	5’-FAM
R	TCTGACTGTCTTCAATTTTTTTTTTTTTTTTTTT TT	
S	TTTTTTTTTTTTTTTTTTTTTAACAGATACATA CGA	
R*	TCGATCTGACTGTCTTCTTTTTTTTTTTTTTTT TTTTTTTTTTTTTTT	T-biotin
S*	TCAGATACATACGACCATTTTTTTTTTTTTTT TTTTTTTTTTTTTTTT	5’-biotin
Z	GTACTGTACGTAGAGTACTTTTTTTTTTTTAT ACATACGACCTATGGTCGTATGTATCTGATT TTTTTTGTGAACTGAGTGTCATAG	
X	CTATGACACTCAGTTCACTTTAGTCCGTGGT	

	AGGGCAGGTTGGGGTGACT	
Y	GGTTGGTGTGGTTGGTTTGTACTCTACGTAC AGTAC	

Table S10. DNA sequences of CAST to regulate GFP gene expression

Name	Sequence	Modification
C3	AATGGTCCTATGATTCCGTTTTTTTTTTTTTG CACGCTGCCGTTACGGCAGCGTGCAGCTCTT TTTTTTATGAGAGAGAGTGTGTCC	
C3-1	AATGGTCCTATGATTCCGTTTTGCACGCTG CCGTTTTTTTTTTTTTTTACGGCAGCGTGCAG CTCATGAGAGAGAGTGTGTCC	
C3-2	AATGGTCCTATGATTCCGTTTTTTTTTGCACG CTGCCGTTTTTTTTTTTTTTTTACGGCAGCGTG CAGCTCTTTTATGAGAGAGAGTGTGTCC	
C3-3	AATGGTCCTATGATTCCGTTTTTTTTTTTTTG CACGCTGCCGTTTTTTTTTTTTTTTACGGCAG CGTGCAGCTCTTTTTTTTATGAGAGAGAGTG TGTCC	
C3-4	AATGGTCCTATGATTCCGTTTTTTTTTGCACG CTGCCGTTACGGCAGCGTGCAGCTCTTTTAT GAGAGAGAG TGTGTCC	
C3-5	AATGGTCCTATGATTCCGTTTTGCACGCTG CCGTTACGGCAGCGTGCAGCTCATGAGAGA GAGTGTGTCC	
D_{GFP}	GAGCTGCACGCTGCCGTCTTTTTTTTTTTTTT TTTTTTTTTTTTTTTTTTTTTTTTTTT	
C3-1	AATGGTCCTATGATTCCGTTTTTTTTTTTTTG CACGCTGCCGTTACGGCAGCGTGCAGCTCTT TTTTTTATGAGAGAGAGTGTGTCC	T-BHQ1
D_{GFP}-1	GAGCTGCACGCTGCCGTCTTTTTTTTTTTTTT TTTTTTTTTTTTTTTTTTTTTTTTTTT	5'-FAM

A	GGACACACTCTCTCTCATTTTAGTCCGTGGTAGGGCAGGTTGGGGTGACT	
B	GGTTGGTGTGGTTGGTTTCGGAATCATAGGACCATT	

Table S11. DNA sequences of CAST to regulate PLK1 gene expression

Name	Sequence	Modification
C4	AATGGTCCTATGATTCCGTTTTTTTTTTTTTACTTGGCAAAGCTTCGGCTTTGCCAAGTGCTTTTTTTTTTATGAGAGAGAGTGTGTCC	
C1	AATGGTCCTATGATTCCGTTTTTTTTTTTTACTTGGCAAAGCTTGCAGTTTTTTTTTTTTTAGTCCGTGGTAGGGCAGGTTGGGGTGACT	
C2	GGTTGGTGTGGTTGGTTTTTTTTTTTTACTGCTTTGGCTTTGCCAAGTGCTTTTTTTTTTATGAGAGAGAGTGTGTCC	
D_{PLK1}	AAGCACTTGGCAAAGCCGCCCTATTCATTCTTCTTGATCCGGTTTTTTTTTTTTTTTTT	
A	GGACACACTCTCTCTCATTTTAGTCCGTGGTAGGGCAGGTTGGGGTGACT	
B	GGTTGGTGTGGTTGGTTTCGGAATCATAGGACCATT	
A1	GGACACACTCTCTCTCATTTTAGTCCGTGGTAGGGCAGGTTGGGGTGACT	T-biotin
B1	GGTTGGTGTGGTTGGTTTCGGAATCATAGGACCATT	T-biotin
A2	GGACACACTCTCTCTCAT	3'-biotin
B2	TCGGAATCATAGGACCATT	5'-biotin

Table S12. DNA sequences of the single-trigger-site CAST triggered by thrombin

**Name**	**Sequence**	**Modification**
C	AATGGTCCTATGATTCCGTTTTTTTTTTTTGTCACTAGCTACTAGGTAGCTAGTGACGTTATTTTTTTTATGAGAGAGAGTGTGTCC	T-BHQ1
D	TAACGTCACTAGCTACCTTTTTTTTTTTTTTTTTTTTTTTTTTTTTTTTTTTTTTTTT	5'-FAM
E-29apt	GGACACACTCTCTCTCATTTTAGTCCGTGGTAGGGCAGGTTGGGGTGACTTTTCGGAATCATAGGACCATT	
E-29apt-1	GGACACACTCTCTCTCATTTTTTTTTAGTCCGTGGTAGGGCAGGTTGGGGTGACTTTTTTTTTCGGAATCATAGGACCATT	
E-29apt-2	GGACACACTCTCTCTCATTTTTTTTTTTTTTTAGTCCGTGGTAGGGCAGGTTGGGGTGACTTTTTTTTTTTTTTCGGAATCATAGGACCATT	
E-29apt-3	GGACACACTCTCTCTCATTTTTTTTTTTTTTTTTTTTTAGTCCGTGGTAGGGCAGGTTGGGGTGACTTTTTTTTTTTTTTTTTTTTTTTCGGAATCATAGGACCATT	
E-29apt-4	GGACACACTCTCTCTCATTTTTTTTTTTTTTTTTTTTTTTTTTTTTTTTTAGTCCGTGGTAGGGCAGGTTGGGGTGACTTTTTTTTTTTTTTTTTTTTTTTTTTTTTTTCGGAATCATAGGACCATT	

Table S13. DNA sequences of the single-trigger-site CAST triggered by PDGF-BB

Name	Sequence	Modification
C	AATGGTCCTATGATTCCGTTTTTTTTTTTTGTCACTAGCTACTAGGTAGCTAGTGACGTTATTTTTTTTATGAGAGAGAGTGTGTCC	
C-1	AATGGTCCTATGATTCCGTTTTTTTTTTTTGTCACTAGCTATAGGTAGCTAGTGACGTTATTTTTTTTATGAGAGAGAGTGTGTCC	
D	TAACGTCACTAGCTACCTTTTTTTTTTTTTTTTTTTTTTTTTTTTTTTTTTTTT	
E-35apt	GGACACACTCTCTCTCATTTTCAGGCTACGGCACGTAGAGCATCACCATGATCCTGTTTCGGAATCATAGGACCATT	
E-43apt	GGACACACTCTCTCTCATTTTACTCAGGGCACTGCAAGCAATTGTGGTCCCAATGGGCTGAGTATTTCGGAATCATAGGACCATT	

References

1. Lin, Y. et al. Ratiometric immunosensor for GP73 detection based on the ratios of electrochemiluminescence and electrochemical signal using DNA tetrahedral nanostructure as the carrier of stable reference signal. Anal. Chem., (2019).
2. Li, L. et al. Aptamer sisplacement reaction from live-cell surfaces and its applications. J. Am. Chem. Soc. 141, 17174-17179 (2019).
3. Dong, H. L. et al. DNA-based reprogramming strategy of receptor-mediated cellular behaviors: from genetic encoding to nongenetic engineering. ACS Appl. Bio Mater. 3, 2796-2804 (2020).
4. Xiong, X. W. et al. Molecular convolutional neural networks with DNA regulatory circuits. Nat. Mach. Intell. 4, 625-635 (2022).
5. Tian, T. R. et al. A framework nucleic acid based robotic nanobee for active targeting therapy. Adv. Funct. Mater. 2007342 (2020).
6. Li, S. P. et al. A DNA nanorobot functions as a cancer therapeutic in response to a molecular trigger *in vivo*. Nat. Biotechnol. 36, 258-264 (2018).
7. Ueki, R. et al. Nongenetic control of receptor signaling dynamics with a DNA-based optochemical tool. Chem. Commun., 57, 5969 (2021).
8. Li, J. et al. Cell-membrane-anchored DNA nanoplatform for programming cellular interactions. J. Am. Chem. Soc. 141, 45, 18013-18020 (2019).
9. Peng, H. et al. A microRNA-initiated DNAzyme motor operating in living cells. Nat. Commun. 8, 14378 (2017).
10. Peng, P. et al. Reconfigurable bioinspired framework nucleic acid nanoplatform dynamically manipulated in living cells for subcellular imaging. Angew. Chem. Int. Ed. 58, 1648-1653 (2019).
11. Wei, J. et al. A smart, autocatalytic, DNAzyme biocircuit for in vivo, amplified, microRNA imaging. Angew. Chem. Int. Ed. 59, 5965-5971 (2020).
12. Yu, Y. J. et al. Intracellular enzyme-powered DNA circuit with a tunable amplifier for miRNA imaging. Chem. Commun. 57, 3753 (2021).
13. Zhang, Q. L. et al. A kinetically controlled platform for ligand-oligonucleotide transduction. Nat. Commun. 12, 4654 (2021).
14. Engelen, W. et al. Antibody-controlled actuation of DNA-based molecular circuits. Nat. Commun. 8, 14473 (2017).
15. Zhang, C. et al. Programmable allosteric DNA regulations for molecular networks and nanomachines. Sci. Adv. 8, eabl4589 (2022).

16. Ranallo, S. et al. Antibody-powered nucleic acid release using a DNA-based nanomachine. Nat. Commun. 8, 15150 (2017).
17. Bracaglia, S. et al. Electrochemical cell-free biosensors for antibody detection. Angew. Chem. Int. Ed. 135, e202216512 (2023).
18. Li, P. et al. Light-driven ATP transmembrane transport controlled by DNA nanomachines. J. Am. Chem. Soc. 140, 16048-16052 (2018).
19. Thompson, L. A. P. et al. Rational design of aptamer switches with programmable pH response. Nat. Commun. 11, 2946 (2020).
20. Xue, C. et al. Programmably tiling rigidified DNA brick on gold nanoparticle as multi-functional shell for cancer-targeted delivery of siRNAs. Nat. Commun. 12, 2928 (2021).
21. Wu, X. H. et al. Genetically encoded DNA origami for gene therapy *in vivo*. J. Am. Chem. Soc. 145, 9343-9353 (2023).
22. Cremers, G. A. O. et al. Determinants of ligand-functionalized DNA nanostructure-cell interactions. J. Am. Chem. Soc. 143, 10131-10142 (2021).
23. Cai, B. et al. Selection of DNA-Encoded Libraries to Protein Targets within and on Living Cells. J. Am. Chem. Soc. 141, 17057-17061 (2019).
24. Jiang, Q. et al. DNA origami as a carrier for circumvention of drug resistance. J. Am. Chem. Soc. 134, 13396-13403 (2012).
25. Zhang, Y. et al. Activating a DNA nanomachine via computation across cancer cell membranes for precise therapy of solid tumors. J. Am. Chem. Soc. 143, 15233-15242 (2021).
26. Di, Z. H. et al. Protease-triggered, spatially controlled DNA assembly in apoptotic cells for early evaluation of therapeutic efficacy. J. Am. Chem. Soc. 145, 7931-7940 (2023).
27. Hong, D. et al. AZD9150, a next-generation antisense oligonucleotide inhibitor of STAT3 with early evidence of clinical activity in lymphoma and lung cancer. Sci. Transl. Med. 7, 314ra185 (2015).
28. Traynor, S. M. et al. Dynamic bio-barcode assay enables electrochemical detection of a cancer biomarker in undiluted human plasma: A sample-in- answer-out approach. Angew. Chem. Int. Ed. 59, 22617-22622 (2020).
29. Koos, B. et al. Proximity-dependent initiation of hybridization chain reaction. Nat. Commun. 6, 7294 (2015).
30. Li, L. et al. Modulating aptamer specificity with pH-responsive DNA bonds. J.

Am. Chem. Soc. 140, 13335-13339 (2018).
31. Li, S. P. et al. A DNA nanorobot functions as a cancer therapeutic in response to a molecular trigger *in vivo*. Nat. Biotechnol. 36, 258-264 (2018).
32. Sun, Y. Y. et al. Nanoscale organization of two-dimensional multimeric pMHC reagents with DNA origami for $CD8^+$ T cell detection. Nat. Commun. 13, 3916 (2022).
33. Shibata, T. et al. Protein-driven RNA nanostructured devices that function *in vitro* and control mammalian cell fate. Nat. Commun. 8, 540 (2017).
34. Wang, Y. J. et al. A biologically stable DNAzyme that efficiently silences gene expression in cells. Nat. Chem. 13, 319-326 (2021).
35. Xiao, M. S. et al. Assembly pathway selection with DNA reaction circuits for programming multiple cell-cell interactions. J. Am. Chem. Soc. 143, 3448-3454 (2021).
36. Li, J. et al. Cell-membrane-anchored DNA nanoplatform for programming cellular interactions. J. Am. Chem. Soc. 141, 45, 18013-18020 (2019).
37. Jiao, K. Programming switchable transcription of topologically constrained DNA. J. Am. Chem. Soc. 142, 10739-10746 (2020).
38. Han, D. et al. Highly specific, single-step cancer cell isolation with multi-aptamer-mediated proximity ligation on live cell membranes. Angew. Chem. Int. Ed. 59, 23564 (2020).
39. Zagorovsky, K. et al. A plasmonic DNAzyme strategy for point-of-care genetic detection of infectious pathogens. Angew. Chem. 125, 3250-3253 (2013).
40. Angerani, S. & Winssinger, N. Sense-and-release logic-gated molecular network responding to dimeric cell surface proteins. J. Am. Chem. Soc. 142, 12333-12340 (2020).
41. Ishaqat, A. et al. Programming DNA circuits for controlled immunostimulation through CpG oligodeoxynucleotide delivery. J. Am. Chem. Soc. (2023).
