# Supplementary material for: Programming conformational cooperativity to regulate allosteric protein-oligonucleotide signal transduction
Source: Nat Commun. 2023 Aug 14;14:4898. doi: 10.1038/s41467-023-40589-z (PMC10425332; doi:10.1038/s41467-023-40589-z)
Supplement: Supplementary file 2 — Reporting Summary [file 41467_2023_40589_MOESM2_ESM.pdf]

nature portfolio

Corresponding author(s): Cheng Zhang, Suping Li

Last updated by author(s): Jul 21, 2023

Reporting Summary

Nature Portfolio wishes to improve the reproducibility of the work that we publish. This form provides structure for consistency and transparency in reporting. For further information on Nature Portfolio policies, see our Editorial Policies and the Editorial Policy Checklist.

Statistics

For all statistical analyses, confirm that the following items are present in the figure legend, table legend, main text, or Methods section.

n/a	Confirmed	
☐	☒	The exact sample size (*n*) for each experimental group/condition, given as a discrete number and unit of measurement
☐	☒	A statement on whether measurements were taken from distinct samples or whether the same sample was measured repeatedly
☐	☒	The statistical test(s) used AND whether they are one- or two-sided<br>*Only common tests should be described solely by name; describe more complex techniques in the Methods section.*
☒	☐	A description of all covariates tested
☐	☒	A description of any assumptions or corrections, such as tests of normality and adjustment for multiple comparisons
☐	☒	A full description of the statistical parameters including central tendency (e.g. means) or other basic estimates (e.g. regression coefficient) AND variation (e.g. standard deviation) or associated estimates of uncertainty (e.g. confidence intervals)
☒	☐	For null hypothesis testing, the test statistic (e.g. *F*, *t*, *r*) with confidence intervals, effect sizes, degrees of freedom and *P* value noted<br>*Give P values as exact values whenever suitable.*
☒	☐	For Bayesian analysis, information on the choice of priors and Markov chain Monte Carlo settings
☒	☐	For hierarchical and complex designs, identification of the appropriate level for tests and full reporting of outcomes
☒	☐	Estimates of effect sizes (e.g. Cohen's *d*, Pearson's *r*), indicating how they were calculated

Our web collection on statistics for biologists contains articles on many of the points above.

Software and code

Policy information about availability of computer code

Data collection	Agilent Technologies (Agilent, USA), ChemiDoc Touch imaging system (Bio-Rad, USA), LSM 710 confocal microscope (Zeiss), Flow cytometry (Thermo Fisher Scientific, USA), NUPACK(NUPACK Web Application, http://www.nupack.org).
Data analysis	AGilent AriaMx 1.8, Image J 1.53c, Prism 7 (GraphPad Software), OriginPro 2018C, FlowJo software package version 10.

For manuscripts utilizing custom algorithms or software that are central to the research but not yet described in published literature, software must be made available to editors and reviewers. We strongly encourage code deposition in a community repository (e.g. GitHub). See the Nature Portfolio guidelines for submitting code & software for further information.

Data

Policy information about availability of data

All manuscripts must include a data availability statement. This statement should provide the following information, where applicable:

- Accession codes, unique identifiers, or web links for publicly available datasets
- A description of any restrictions on data availability
- For clinical datasets or third party data, please ensure that the statement adheres to our policy

Source data are provided as a Source Data file.

Human research participants

Policy information about studies involving human research participants and Sex and Gender in Research.

Reporting on sex and gender	not applicable
Population characteristics	not applicable
Recruitment	not applicable
Ethics oversight	not applicable

Note that full information on the approval of the study protocol must also be provided in the manuscript.

Field-specific reporting

Please select the one below that is the best fit for your research. If you are not sure, read the appropriate sections before making your selection.

☒ Life sciences ☐ Behavioural & social sciences ☐ Ecological, evolutionary & environmental sciences

For a reference copy of the document with all sections, see nature.com/documents/nr-reporting-summary-flat.pdf

Life sciences study design

All studies must disclose on these points even when the disclosure is negative.

Sample size	Sample size was determined based on standards for experimental cell biology, attempting to have a minimum of N = 3 biological replicates with sufficient reproducibility.
Data exclusions	All collected data from planned experiments were included in the analyses.
Replication	All experimental findings were replicated at least 3 times with enough reproducibility. Therefore, the attempts of data replication were successful.
Randomization	As we performed experiments with defined genetic and technique background, there were no need for randomization.
Blinding	In general, investigators were blinded during experimental execution and data collection. We always set control and repeat experiment group for this study. Determinations of relevant parameters are considered objective measurements that are not subject to bias, so the integrity of the results is not compromised when the study and unblinded analysis are performed.

Reporting for specific materials, systems and methods

We require information from authors about some types of materials, experimental systems and methods used in many studies. Here, indicate whether each material, system or method listed is relevant to your study. If you are not sure if a list item applies to your research, read the appropriate section before selecting a response.

Materials & experimental systems

n/a	Involved in the study	
☐	☒	Antibodies
☐	☒	Eukaryotic cell lines
☒	☐	Palaeontology and archaeology
☒	☐	Animals and other organisms
☒	☐	Clinical data
☒	☐	Dual use research of concern

Methods

n/a	Involved in the study	
☒	☐	ChIP-seq
☐	☒	Flow cytometry
☒	☐	MRI-based neuroimaging

Antibodies

Antibodies used	Primary antibodies against PLK1 (EPR19534(ab189139)) and GAPDH (6C5(ab8245)) were purchased from Abcam (Cambridge, UK). HRP-linked goat anti-rabbit secondary antibodies (PR30011) were purchased from Proteintech (Wuhan, China).

Validation	Anti-PLK1 antibody source: Rabbit. Species Reactivity: Human. Suitable for: Flow Cyt (Intra), ICC/IF, IP, WB.<br>Anti-GAPDH antibody source: Mouse. Species Reactivity: Mouse, Rat, Human. Suitable for: ICC/IF, WB.

Eukaryotic cell lines

Policy information about cell lines and Sex and Gender in Research

Cell line source(s)	The human cervical carcinoma cell line (HeLa) was purchased from the American Type Culture Collection (ATCC, FS-0252).
Authentication	All cell lines were authenticated by STR profiling.
Mycoplasma contamination	All cells were tested for mycoplasma contamination and had no mycoplasma contamination.
Commonly misidentified lines (See ICLAC register)	None of the cell lines used are classified as commonly misidentified lines.

Flow Cytometry

Plots

Confirm that:

- [x] The axis labels state the marker and fluorochrome used (e.g. CD4-FITC).
- [x] The axis scales are clearly visible. Include numbers along axes only for bottom left plot of group (a 'group' is an analysis of identical markers).
- [x] All plots are contour plots with outliers or pseudocolor plots.
- [x] A numerical value for number of cells or percentage (with statistics) is provided.

Methodology

Sample preparation	The HeLa cells were incubated with Cy5-labeled nucleic acid strands for 3-4 h at 37°C.
Instrument	Thermo Fisher Scientific Attune NxT Flow Cytometer was used for data collection in our study.
Software	Data were analyzed via the FlowJo software package version 10 (FlowJo LLC, Ashland, OR, USA).
Cell population abundance	At least 10,000 events were recorded during the sort for setting the sorting criteria and for post-sort analysis.Post-sort fractions had higher than 95% purify, as verified by flow cytometry analysis on the same machine used to sort the cells.
Gating strategy	H Gates was used to exclude debris and cell aggregates in FSC-A/SSC-A and FSC-A/FSC-H plots,and fluorescence quadrant gate was chosen to discriminate between "Cy5-positive"and "Cy5-negative" cells.

- [x] Tick this box to confirm that a figure exemplifying the gating strategy is provided in the Supplementary Information.
